# Predicting lifetime cardiovascular risk and benefits of preventive treatment in patients with established atherosclerotic cardiovascular disease: the SMART-REACH2 model

**DOI:** 10.1093/eurheartj/ehag400

**Published:** 2026-06-05

**Authors:** Joris Holtrop, Mari N Gynnild, Salil V Deo, Carl-Emil Lim, Stelios Boulitsakis Logothetis, Kristi Läll, Minna Oksanen, Håvard Dalen, John Munkhaugen, Peter Ueda, Tomas Jernberg, José Tuñón, Ignacio Mahíllo Fernández, Ingvild Saltvedt, Hanne Ellekjær, Martin Teraa, Manon G van der Meer, Ynte M Ruigrok, Urmo Võsa, Małgorzata Chlabicz, Karol Kaminski, Ph Gabriel Steg, Ivana Burazor, Reedik Mägi, Taavi Tillmann, David A Wood, Kausik K Ray, Dirk de Bacquer, Janne Martikainen, John William McEvoy, Stephen Kaptoge, Frank L J Visseren, Emanuele Di Angelantonio, Deepak L Bhatt, Angela Wood, Steven H J Hageman, Jannick A N Dorresteijn

**Affiliations:** Department of Vascular Medicine and Endocrinology, University Medical Centre Utrecht, Utrecht, The Netherlands; Clinic of Cardiology, St.Olav University Hospital, Trondheim, Norway; K.G. Jebsen Center for Cardiac Biomarkers, Institute of Clinical Medicine, University of Oslo, Oslo, Norway; Department of Circulation and Medical Imaging, Faculty of Medicine and Health Science, NTNU – Norwegian University of Science and Technology, Trondheim, Norway; Surgical Services, Louis Stokes Cleveland VA Medical Center, Cleveland, OH, USA; Case School of Medicine, Case Western Reserve University School of Medicine, Cleveland, OH, USA; School of Health and Wellbeing, University of Glasgow, Glasgow, UK; Clinical Epidemiology Division, Department of Medicine, Karolinska Institutet, Stockholm, Sweden; British Heart Foundation Cardiovascular Epidemiology Unit, Department of Public Health and Primary Care, University of Cambridge, Cambridge, UK; Victor Phillip Dahdaleh Heart and Lung Research Institute, University of Cambridge, Cambridge, UK; Estonian Genome Centre, Institute of Genomics, University of Tartu, Tartu, Estonia; ESiOR Oy, Kuopio, Finland; Clinic of Cardiology, St.Olav University Hospital, Trondheim, Norway; Department of Circulation and Medical Imaging, Norwegian University of Science and Technology, Trondheim, Norway; Department of Stroke, Clinic of Medicine, St.Olav University Hospital, Trondheim, Norway; Department of Medicine, Levanger Hospital, Nord- Trøndelag Hospital Trust, Levanger, Norway; Department of Behavioral Medicine, Faculty of Medicine, University of Oslo, Oslo, Norway; Department of Medicine, Drammen Hospital, Vestre Viken Trust, Drammen, Norway; Clinical Epidemiology Division, Department of Medicine, Karolinska Institutet, Stockholm, Sweden; Department of Clinical Sciences, Cardiology, Karolinska Institutet Danderyd Hospital, Stockholm, Sweden; Cardiology Department, Fundacion Jimenez Diaz University Hospital, Autónoma University and CIBERCV, Madrid, Spain; Biostatistics and Epidemiology Unit, Health Research Institute-Fundación Jiménez Díaz University Hospital, Universidad Autónoma de Madrid, Madrid, Spain; Department of Neuromedicine and Movement Science, Faculty of Medicine and Health Science, NTNU – Norwegian University of Science and Technology, Trondheim, Norway; Department of Geriatric Medicine, St.Olavs Hospital, Trondheim University Hospital, Trondheim, Norway; Department of Stroke, Clinic of Medicine, St.Olav University Hospital, Trondheim, Norway; Department of Neuromedicine and Movement Science, Faculty of Medicine and Health Science, NTNU – Norwegian University of Science and Technology, Trondheim, Norway; Department of Vascular Surgery, University Medical Center Utrecht, Utrecht, The Netherlands; Department of Cardiology, University Medical Center Utrecht, Utrecht, The Netherlands; Department of Neurology and Neurosurgery, University Medical Center Utrecht, Utrecht, The Netherlands; Estonian Genome Centre, Institute of Genomics, University of Tartu, Tartu, Estonia; Department of Population Medicine and Lifestyle Diseases Prevention, Medical University of Białystok, Białystok, Poland; Department of Invasive Cardiology, Medical University of Białystok, Białystok, Poland; Department of Population Medicine and Lifestyle Diseases Prevention, Medical University of Białystok, Białystok, Poland; Department of Cardiology and Internal Diseases, Medical University of Białystok, Białystok, Poland; Université Paris-Cité, FACT, French Alliance for Cardiovascular Trials, AP-HP, Hôpital Bichat, and INSERM U-1148, Paris, France; Faculty of Medicine, Institute for Cardiovascular Diseases ‘Dedinje’ and Belgrade University, Belgrade, Serbia; Estonian Genome Centre, Institute of Genomics, University of Tartu, Tartu, Estonia; Institute of Family Medicine and Public Health, University of Tartu, Tartu, Estonia; National Institute for Prevention and Cardiovascular Health, University of Galway, Galway, Republic of Ireland; School of Public Health, Imperial College London, London, UK; Department of Public Health and Primary Care, Ghent University, Gent, Belgium; ESiOR Oy, Kuopio, Finland; School of Pharmacy, Faculty of Health Sciences, Kuopio Campus, University of Eastern Finland, Kuopio, Finland; National Institute for Prevention and Cardiovascular Health, University of Galway, Galway, Republic of Ireland; British Heart Foundation Cardiovascular Epidemiology Unit, Department of Public Health and Primary Care, University of Cambridge, Cambridge, UK; Victor Phillip Dahdaleh Heart and Lung Research Institute, University of Cambridge, Cambridge, UK; Department of Vascular Medicine and Endocrinology, University Medical Centre Utrecht, Utrecht, The Netherlands; British Heart Foundation Cardiovascular Epidemiology Unit, Department of Public Health and Primary Care, University of Cambridge, Cambridge, UK; Victor Phillip Dahdaleh Heart and Lung Research Institute, University of Cambridge, Cambridge, UK; British Heart Foundation Centre of Research Excellence, University of Cambridge, Cambridge, UK; National Institute for Health and Care Research Blood and Transplant Research Unit in Donor Health and Behaviour, University of Cambridge, Cambridge, UK; Health Data Research UK Cambridge, Wellcome Genome Campus and University of Cambridge, Cambridge, UK; Cambridge Centre of Artificial Intelligence in Medicine, Cambridge, UK; British Heart Foundation Data Science Centre, Health Data Research UK, London, UK; Mount Sinai Fuster Heart Hospital, Icahn School of Medicine at Mount Sinai, NewYork, NY, USA; British Heart Foundation Cardiovascular Epidemiology Unit, Department of Public Health and Primary Care, University of Cambridge, Cambridge, UK; Victor Phillip Dahdaleh Heart and Lung Research Institute, University of Cambridge, Cambridge, UK; British Heart Foundation Centre of Research Excellence, University of Cambridge, Cambridge, UK; National Institute for Health and Care Research Blood and Transplant Research Unit in Donor Health and Behaviour, University of Cambridge, Cambridge, UK; Health Data Research UK Cambridge, Wellcome Genome Campus and University of Cambridge, Cambridge, UK; Cambridge Centre of Artificial Intelligence in Medicine, Cambridge, UK; British Heart Foundation Data Science Centre, Health Data Research UK, London, UK; Department of Vascular Medicine and Endocrinology, University Medical Centre Utrecht, Utrecht, The Netherlands; Department of Vascular Medicine and Endocrinology, University Medical Centre Utrecht, Utrecht, The Netherlands

**Keywords:** Established atherosclerotic cardiovascular disease, Personalized treatment, Recurrent risk, Residual risk, Risk prediction, Secondary prevention

## Abstract

**Background and Aims:**

The 2021 ESC guidelines on cardiovascular (CV) disease prevention recommend the SMART-REACH lifetime risk model to guide treatment decisions in patients with established atherosclerotic CV disease. The aim was to develop the SMART-REACH2 model for estimating lifetime risk of recurrent CV events and treatment benefits in patients with established atherosclerotic CV disease, with systematic recalibration to the four European and other global risk regions.

**Methods:**

SMART-REACH2 was derived in 8708 individuals aged 40–90 years with coronary, cerebrovascular, peripheral artery disease and/or abdominal aortic aneurysm from the UCC-SMART cohort. Sex-stratified, cause-specific Cox models for recurrent CV events and non-CV death were fitted using age as timescale and routinely available predictors. Recurrent CV events were defined as a composite of myocardial infarction, stroke, or CV death. Recalibration was based on representative cohorts per risk region. External validation was performed in 2 085 780 patients from 54 countries; model performance was assessed by calibration plots and Harrell's C-statistic.

**Results:**

In the derivation cohort, 2057 recurrent CV events occurred over a median follow-up of 8.5 years (25th–75th: 4.3–13.0). In external validation, 307 706 events occurred. The pooled C-statistic was 0.68 (95% confidence interval 0.66–0.69) and ranged from 0.66 (0.64–0.69) for European low-risk region up to 0.72 (0.66–0.78) for Latin America, with adequate calibration across risk regions. Performance was consistent across sexes and CV disease subtypes. Using SMART-REACH2, estimated potential gains in CV disease-free life expectancy for a 50-year-old example patient receiving intensified preventive treatment (15 mmHg systolic blood pressure and 1.0 mmol/L low-density lipoprotein cholesterol reduction) ranged from 2 years in the low-risk region to 4.4 years in the very-high-risk region.

**Conclusions:**

The updated SMART-REACH2 model accounts for geographical and sex-specific variations and allows estimation of short-term and lifetime risk of recurrent CV events and treatment benefits, facilitating shared decision-making as recommended by guidelines.

## Structured Graphical Abstract

**Graphical Abstract:**
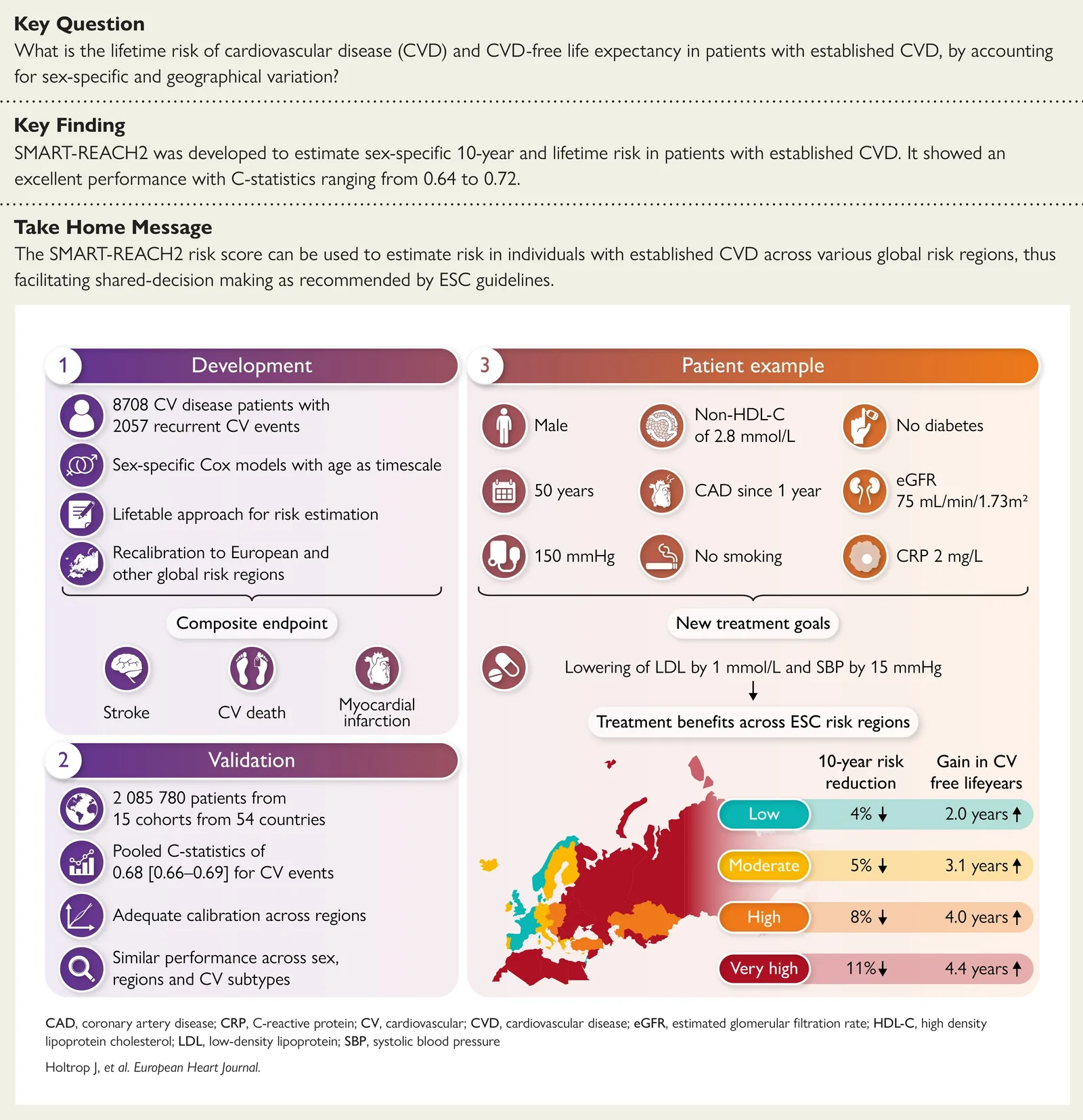
Created with BioRender.com

**See the editorial comment for this article ‘Improving individual lifetime cardiovascular disease risk estimation to optimize secondary prevention’, by D. Mukherjee and S. Mani, https://doi.org/10.1093/eurheartj/ehag479.**

## Introduction

While patients with established atherosclerotic cardiovascular (CV) disease are considered to be at high risk of recurrent CV events, their individual risk can vary substantially.^1^ To navigate this variation, guidelines recommend the use of risk prediction models estimating 10-year or lifetime risk of recurrent CV events to identify those patients at high risk or with potentially high treatment benefits.^2^ As age is a major driver of CV risk, short-term prediction models tend to identify older patients, whereas lifetime models identify younger patients at high risk, thereby facilitating timely initiation of (cost-)effective treatments.^2–4^ Lifetime models can express benefits of preventive treatment in terms of individual potential CV disease-free life-years gained due to treatment initiation or intensification. Such estimates are easily comprehensible for patients and may improve patient engagement, adherence, and risk factor control.^5,6^ The 2021 ESC Guidelines on CV disease prevention in clinical practice recommend the SMART-REACH model for estimating the lifetime risk of recurrent CV events in patients with established atherosclerotic CV disease.^2,7^ The SMART-REACH model is implemented as an interactive calculator in the ESC CV Disease Risk Calculation-app and the CE-marked U-Prevent.com (https://u-prevent.com/) medical device.

Despite its strengths, the SMART-REACH model has potential for improvement in the context of contemporary clinical practice. Firstly, SMART-REACH accounts for some variation in CV disease background risk, but not at the finer regional level of the European risk regions as defined by the ESC and used by models such as SCORE2.^2^ Second, sex-specific development might lead to improved risk prediction in men and women.^8^ The current study aims to address this through the use of contemporary data and sex-specific development of the SMART-REACH2 model for estimating lifetime risk in patients aged 40–90 years with established atherosclerotic CV disease, and through its systematic recalibration to the four European and other global risk regions.

## Methods

### Model development

The SMART-REACH2 model was developed using a two-step approach. First, the base of the model was derived using data from patients aged 40–90 years with established atherosclerotic CV disease in the Utrecht Cardiovascular Cohort (UCC-SMART). The UCC-SMART cohort is an ongoing single-centre prospective cohort study at the University Medical Centre Utrecht, The Netherlands. Details have been described previously.^9^ Briefly, since 1996 patients newly referred to the University Medical Centre Utrecht with, or at high risk for, CV disease can be included. Baseline characteristics were determined using a standardized baseline assessment protocol consisting of questionnaires, physical examination, and laboratory testing. To negate the effects of the COVID-19 pandemic, follow-up time was truncated at 1 February 2020, and analyses were restricted to patients included before this date (*n* = 8708). Established atherosclerotic CV disease at baseline was defined as coronary artery disease (CAD), cerebrovascular disease (CeVD), peripheral artery disease (PAD), and/or abdominal aortic aneurysm (AAA) (see Supplementary data online, *Table S1* for definitions).

The second step in model development was the incorporation of recalibration factors to account for regional variations in background CV risk, thus ensuring that the model can be applied across these regions. These regions where the ESC-defined European risk regions (low-, moderate-, high-, and very-high-risk), which are based on CV disease-related mortality rates from the World Health Organization (see Supplementary data online, *Figure S1*). In the absence of similar classifications, regions outside Europe were grouped based on geography. The recalibration factors for each of these regions are sex-specific expected-observed ratios for the separate endpoints. These were derived from the cohorts deemed most representative for reach risk region considering their size and broad patient inclusion. For the European high- and very-high-risk regions the cohorts within each respective risk region were combined, due to limited number of events in individual cohorts available for recalibration. The cohorts used for recalibration were CPRD for the European low-risk region, SWEDEHEART for the European moderate-risk region, the Estonian Biobank for the European high-risk region, and the combination of EUROASPIRE V, REACH and SURF CHD II for the European very-high-risk region. For other global regions, REACH was used for recalibration, except for the VA which was recalibrated independently. Methods for recalibration for future data sources are available in Supplementary data online, Methods.

### External validation

Including these cohorts used for recalibration a total of 15 cohorts with patients aged 40–90 with established atherosclerotic CV disease were available for external validation. Cohorts spanning multiple risk regions were analysed separately by region. For the European low-risk region, the validation cohorts were the United Kingdom's Clinical Practice Research Datalink (CPRD, *n* = 147 566) the National Health Service in England Secure Data Environment accessed via the British Heart Foundation Data Science Centre's CVD-COVID-UK/COVID-IMPACT Consortium (https://bhfdatasciencecentre.org/areas/cvd-covid-uk-covid-impact/) (NHSE SDE, *n* = 931 460), the Norwegian Trøndelag Health Study (HUNT3, *n* = 5698 and HUNT4, *n* = 6010), the Norwegian COgnitive impairment After STroke (Nor-COAST, *n* = 654), and NORwegian CORonary Prevention (NORCOR, *n* = 1112) cohorts, the Spanish Biomarkers in Acute Coronary Syndrome and Biomarkers in Acute Myocardial Infarction (BACS/BAMI, *n* = 946), and data from low-risk countries in the international Reduction of Atherothrombosis for Continued Health (REACH, *n* = 6616) registry and European Action on Secondary and Primary Prevention by Intervention to Reduce Events (EUROASPIRE V, *n* = 1082) survey. The moderate-risk region validation cohorts included the Swedish Web-System for Enhancement and Development of Evidence-Based Care in Heart Disease Evaluated According to Recommended Therapies (SWEDEHEART, *n* = 87 847), the North Karelia Wellbeing Services County cohort from Finland (Karelia, *n* = 3303), and moderate-risk countries from REACH (*n* = 6266) and EUROASPIRE V (*n* = 1723). The high-risk region validation cohorts comprised the Estonian Biobank (*n* = 11 669), the Polish Bialystok PLUS/Polaspire (POLASPIRE, *n* = 243), and high-risk countries from REACH (*n* = 836) and EUROASPIRE V (*n* = 1529). For the very-high-risk region validation cohorts were the Serbian part of the Survey of Risk Factors in Coronary Heart Disease (SURF CHD) II registry (*n* = 203), and very-high-risk countries from REACH (*n* = 4382) and EUROASPIRE V (*n* = 2710). Validation cohorts outside of Europe included the American Veterans Health Administration (VA, *n* = 930 725) and non-European parts of REACH (*n* = 28 407) which were subdivided into North America (*n* = 15 857), Latin America (*n* = 1446), Australia (*n* = 1963), Middle Eastern countries (*n* = 654), and Asian countries (*n* = 4742) excluding Japan (*n* = 3745) due to differences in CV risk. In total the 15 external validation cohorts covered 54 countries.

Definitions of established atherosclerotic CV disease at baseline in the external validation cohorts were aligned as closely as possible with the definitions in the derivation data. Baseline presence of atherosclerotic CV disease was ascertained through either (i) questionnaires/records or (ii) linkage to national databases (CPRD, NHSE SDE, HUNT3, HUNT4, SWEDEHEART, Karelia, Estonian Biobank). International Classification of Diseases (ICD) codes for disease history are available in Supplementary data online, *Table S2*. Outcomes were ascertained by (i) linkage to primary care records, hospital records, or disease/mortality registries, (ii) contacting patients (SURF CHD, POLASPIRE), annual questionnaires (REACH registry), or combination of these (EUROASPIRE V). More detailed descriptions of these cohorts are provided in Supplementary data online, Methods, with additional information available in prior publications.^10–26^ For the main analyses, follow-up and inclusion were restricted to a period prior to the COVID-19 pandemic if applicable. A sensitivity analysis regarding performance of SMART-REACH2 during the COVID-19 pandemic was performed in a second NHSE SDE cohort spanning including patients with established CV disease alive on 1 January 2020 and follow-up until 1 March 2022 (*n* = 878 660).

### Outcome

The primary outcome of SMART-REACH2 was the same as in the original SMART-REACH model: recurrent CV events, defined as a composite of myocardial infarction, CeVD, or CV death.^7^ Death from causes not included in CV death (i.e. non-CV death) was considered the competing endpoint. The ICD codes used for endpoint definitions in the external validation cohorts are available in Supplementary data online, *Table S3*.

### Statistical analyses

Sex-stratified, cause-specific Cox proportional hazard models were developed separately for recurrent CV events and non-CV death. Age was used as the underlying timescale, with age at entry into the cohort as the start of follow-up (i.e. left truncation) up to age of censoring (end of follow-up) or (primary or competing) event (i.e. right censoring). To adjust for any effect of age of entry in the cohort, age was added as a predictor.^27^ All models included the following prespecified predictors: age at entry, current smoking, separate indicators for history of CAD, CeVD, PAD, and AAA, diabetes mellitus (any type), (office) systolic blood pressure (SBP), non-high-density lipoprotein (HDL; mmol/L) cholesterol, years since first atherosclerotic CV disease diagnosis at enrolment (those defining the study population), estimated glomerular filtration rate (eGFR) as estimated by the Chronic Kidney Disease Epidemiology Collaboration Epidemiology Collaboration equations and C-reactive protein (CRP; mg/L). For the model predicting recurrent CV events, use of any type of antithrombotic medication was included as an offset term with a hazard ratio (HR) of 0.81.^28,29^ This offset term promotes both transferability to other settings by limiting the influence of local prescription practices, and clinical utility by resulting in risk estimates that can inform future treatment decisions on antithrombotic treatment (e.g. initiation of dual-pathway inhibition).^28^

To limit influence of outliers on model parameters, continuous predictors were winsorized at their sex-specific 1st and 99th percentiles. The need for non-linear transformations was assessed for each continuous predictor by comparing Akaike's Information Criterion between models with and without squared polynomials, cubic polynomials, logarithmic transformations, and square root transformations. Proportional hazards were assessed through visual inspection of Schoenfeld residuals. If applicable, interactions with age were considered. No time-varying covariates were incorporated. To decrease the odds of chance findings, non-linear transformations were initially assessed in the combined population before proceeding with the sex-specific development. Ridge penalization using penalized maximum likelihood estimation was applied to reduce optimism. Optimal shrinkage was determined using five-fold cross-validation with identical folds for both endpoints to ensure proportional congruence. Continuous predictors were centred and scaled (using means and standard deviations, respectively) to enable proportionate penalization (see Supplementary data online, Methods).

Baseline risks were derived by estimating 1-year interval endpoint-specific survival from the cause-specific Cox models over the complete age range (40–90 years), using a reference individual with sex-specific mean values for all risk factors and absence of binary predictors and keeping the risk factor profile stable. These intervals were smoothed using an exponential model weighted by the number of patients in each age group and bounded at 100% survival probability to ensure realistic probabilities (see Supplementary data online, Methods).

Missing data were handled using the *mice* package, with predictive mean matching for continuous predictors and logistic regression for the binary predictors (15 datasets and 50 iterations). Both endpoints were incorporated using Nelson-Aalen estimates.^30^ Convergence of the imputation model was assessed using trace plots. Estimates were pooled using Rubin's rules where applicable. Missingness per predictor variable in the derivation data is available in Supplementary data online, *Table S4*. Missing values in the external validation cohorts were handled within each cohort separately (see Supplementary data online, Methods).^31^

### Prediction of individual and lifetime risk

Lifetime risk was defined as a patient's cumulative risk of experiencing a recurrent CV event from their current age up to age 90. Risks were estimated using the lifetable approach which has been validated for prediction horizons up to at least 17 years.^31^ The 1-year risks of recurrent CV events and non-CV death were calculated based on a patient's risk factors for each remaining life year up to age 90. The probability of surviving each year event-free (interval survival probability) was estimated, accounting for the competing risks between CV events and non-CV death. These interval survival probabilities were multiplied to calculate the cumulative probability of reaching a specific age free of any event. CV disease-free life expectancy was defined as the age at which this cumulative survival probability drops below 50%. A detailed example of the lifetable calculations is provided in Supplementary data online, Methods. An interactive online version of the SMART-REACH2 will be made available until future implementation of the model for clinical use in U-prevent.com.

### Model performance

Model performance was assessed in terms of discrimination and calibration. Discrimination was assessed using Harrel's concordance (C)-statistic and calibration was assessed through calibration plots. Competing risk-adjusted C-statistics were pooled using a random effects model with inverse variance weighting to account for differences such as follow-up and event rates impacting precision.^32^ Calibration was evaluated visually through calibration plots of predicted versus observed risks, which were adjusted for competing risks (see Supplementary data online, Methods). Age-specific calibration plots were constructed to assess model performance across observed ages and provide insight into the risk accumulation approach underlying lifetime estimates. Cumulative incidence curves were used to determine the maximum follow-up duration at which model performance could be evaluated in each cohort, with a maximum of 10 years of follow-up. The follow-up times at which model performance was assessed were 2 years for Nor-COAST, HUNT4, both NHSE SDE cohorts, the combined high- and very-high-risk region cohorts, and all REACH regional sub cohorts; 3 years for the VA and the Karelia Health Service cohort; 6 years for BACS/BAMI; 7 years for NORCOR; 8 years for the Estonian Biobank, and CPRD; and 10 years for SWEDEHEART and HUNT3. REACH data from Middle Eastern and Asian countries (excluding Japan) were combined due to comparable event rates and limited cohort sizes, and performance was assessed at 2-year follow-up. The SMART-REACH2 model was compared to the original SMART-REACH model in the HUNT3 cohort and the SWEDHEART registry to assess the impact of the regional recalibration. Performance was assessed through C-statistics, calibration, and clinical utility assessed through decision curve analyses.

Performance was assessed in the overall population and separately in men and women. An additional analysis was performed for the VA, restricting to those eligible only for healthcare through the VA (*n* = 103 439) to assess the potential for missing outcomes. Two sensitivity analyses were performed. First, to assess the performance across CV disease subtypes (i.e. CAD, CeVD, and PAD/AAA), and their respective (heterogeneous) aetiologies, discrimination and calibration were assessed in two scenarios: (i) among all patients with a specific CV disease subtype regardless of concomitant CV subtypes (e.g. all patients with CeVD, including those with additional CV subtypes) and (ii) among patients with only a single CV disease subtype (e.g. patients with only CeVD, without CAD or PAD/AAA). For the first scenario, discrimination was assessed in all cohorts, the second scenario was only assessed in HUNT3 where most CV subtypes were represented, and follow-up was long. Calibration for both scenarios was assessed in HUNT3 only. The sensitivity analyses regarding performance during the COVID-19 pandemic was assessed in the NHSE SDE pandemic-specific cohort, and these results were not included in the pooled performance estimates.

### Estimation of treatment effects

One potential application of the SMART-REACH2 model is the estimation of individual benefits from initiation or intensification of preventive treatment, similar to the original SMART-REACH model.^7,31^ Such benefits can be estimated by combining the risk predictions from the SMART-REACH2 model with relative treatment effects derived from clinical trials or meta-analyses, as detailed in Supplementary data online, Methods.^33^ This approach parallels other proposed methods of integrating treatment effects with risk prediction to estimate individual benefits.^34^ As an illustrative example, the SMART-REACH2 model was combined with the effect of lowering of SBP (HR 0.80 per 10 mmHg reduction) and low-density lipoprotein (LDL) cholesterol (HR 0.78 per 1 mmol/L reduction) as obtained from meta-analyses.^35,36^ These effects were applied to four identical example patients from all four European risk regions in order to estimate the benefits of these preventive treatments. These benefits can be expressed as absolute risk reductions and gain in CV disease-free life expectancy as a result of treatment. Assumptions for this approach are listed in Supplementary data online, Methods. All analyses were performed with R-statistic programming Version (4.4.1). Results are reported in accordance with TRIPOD (see Supplementary data online, Checklist).

## Results

### Model development

The derivation cohort consisted of 8708 patients with established atherosclerotic CV disease from UCC-SMART. Mean age at baseline was 61 ± 9 years and 26% (*n* = 2257) were women. CAD was the most prevalent atherosclerotic CV disease subtype (62%; *n* = 5412), followed by CeVD (29%; *n* = 2527), PAD (18%; *n* = 1534), and AAA (8%; *n* = 729). Overall, 15% (*n* = 1318) had two or more atherosclerotic CV disease subtypes. Baseline characteristics stratified by sex are available in *Table 1*.

**Table 1:** Baseline characteristics of the UCC-SMART cohort according to sex

	Women (*n* = 2257)	Men (*n* = 6451)
Age (years)	61 ± 10	62 ± 9
Current smoking	33 (741)	29 (1840)
Systolic blood pressure (mmHg)	137 [124–151]	137 [125–150]
BMI (kg/m^2^)	26 [23–29]	27 [24–29]
C-reactive protein (mg/L)	2.2 [1.0–4.7]	1.9 [0.9–4.0]
eGFR (mL/min/1.73 m^2^)	75 [62–87]	79 [66–90]
Total cholesterol (mmol/L)	4.9 [4.2–5.9]	4.5 [3.8–5.4]
HDL cholesterol (mmol/L)	1.4 [1.1–1.7]	1.1 [0.9–1.3]
LDL cholesterol (mmol/L)	2.8 [2.1–3.6]	2.6 [2.0–3.4]
Non-HDL cholesterol (mmol/L)	3.5 [2.7–4.4]	3.3 [2.7–4.2]
Triglycerides (mmol/L)	1.4 [1.0–1.9]	1.4 [1.0–2.0]
Diabetes mellitus	16 (369)	18 (1156)
History of coronary artery disease	45 (1020)	68 (4392)
History of cerebrovascular disease	40 (913)	25 (1614)
History of peripheral artery disease	22 (505)	16 (1029)
History of abdominal aortic aneurysm	5.2 (117)	9.5 (612)
Years since first atherosclerotic CV disease diagnosis	0 [0–2]	1 [0–5]
Use of antithrombotic medication	78 (1759)	86 (5571)
Use of lipid-lowering medication	63 (1423)	73 (4681)
Use of blood pressure-lowering medication	71 (1610)	78 (5007)
Follow-up (years)	8.8 [4.7–13.4]	8.3 [4.2–12.9]
Incidence of recurrent CV events	19 (436)	25 (1621)
Rate of recurrent CV events/1000 PY [95% CI]	21 [19–23]	28 [27–30]
Incidence of non-CV death	16 (367)	18 (1131)
Rate of non-CV death/1000 PY [95% CI]	18 [16–20]	20 [19–21]

Data are presented as % (*n*), mean ± standard deviation, or median [25th–75th percentile] as appropriate.

BMI, body mass index; CI, confidence interval; CV, cardiovascular; eGFR, estimated glomerular filtration rate (calculated with the Chronic Kidney Disease Epidemiology Collaboration formula); HDL, high-density lipoprotein; LDL, low-density lipoprotein; PY, person-years.

The event rates are not adjusted for competing risks as the models are developed endpoint specific.

Over a median follow-up of 8.5 [25th–75th percentile range; 4.3–13.0] years, 2057 recurrent CV events and 1498 non-CV deaths occurred. HRs for each predictor per endpoint are provided in Supplementary data online, *Table S5*. No violations of the proportional hazards were observed and no interactions with age were required. The unsmoothed 1-year interval survivals and fitted exponential models are available in Supplementary data online, *Figures S3* and *S4*. For the base model, the C-statistic for recurrent CV events upon internal validation was 0.72 [0.68–0.71] and for non-CV death 0.72 [0.70–0.73]. Calibration at 10 years was adequate for both endpoints (see Supplementary data online, *Figure S5*). The regional recalibration factors incorporated onto the base model are available in Supplementary data online, *Table S9*. All parameters required for risk estimation using SMART-REACH2 are available in Supplementary data online, *Tables S6–9*, including a worked-out example (see Supplementary data online, *Figure S2*). R code for the SMART-REACH2 model with illustration of use are available on GitHub (https://github.com/JorisHolt/SMARTREACH2). An online interactive calculator for the SMART-REACH2 model is available here (https://holtropjoris.shinyapps.io/SMART-REACH2/).

### External validation

External validation was performed in 2 085 780 patients, among whom 396 902 women, with established atherosclerotic CV disease from 15 cohorts spanning 54 countries. Mean age at baseline was 69 ± 10 years. Most data (58%, *n* = 1 206 351) originated from cohorts in Europe. Median follow-up ranged from 1.3 [1.1–1.6] years in EUROASPIRE-V to 12.0 [5.8–13.8] years in HUNT3. In total, 307 706 recurrent CV events and 91 967 non-CV deaths occurred during follow-up. The incidence rate for recurrent CV events varied from 24 [95% confidence interval: 19–29] to 100 [100–100] per 1000 person-years between cohorts (*Table 2*). The incidence rate of non-CV death varied from 3 [0–9] to 29 [21–40] per 1000 person-years between cohorts (*Table 2*).

**Table 2:** Baseline characteristics of the external validation cohorts

	CPRD	NHSE SDE	HUNT3	HUNT4	NORCOR	Nor-COAST	BACS/BAMI	SWEDEHEART
Country	UK	UK	Norway	Norway	Norway	Norway	Spain	Sweden
European risk region	Low risk	Low risk	Low risk	Low risk	Low risk	Low risk	Low risk	Moderate risk
Number of patients	147 566	931 460	5698	6046	1112	654	946	87 847
Age (years)	71 ± 10	66 ± 9	69 ± 11	70 ± 10	62 ± 9	73 ± 10	62 ± 12	64 ± 9
Male sex	59 (87 554)	70 (656 125)	57 (3249)	58 (3486)	79 (879)	42 (274)	76 (720)	74 (64 759)
Current smoking	24 (34 956)	20 (181 400)	23 (1291)	13 (786)	21 (233)	9.8 (64)	13 (127)	12 (10 156)
Systolic blood pressure (mmHg)	137 [125–146]	130 [120–140]	135 [122–148]	134 [121–147]	136 [125–150]	140 [128–153]	130 [120–140]	130 [120–140]
BMI (kg/m^2^)	28 [24–3]	29 [25–33]	-	-	28 [26–31]	-	28 [26–31]	27 [25–30]
C-reactive protein (mg/L)	-	-	1.6 [0.8–3.5]	1.3 [0.7–2.7]	1.6 [0.8–2.9]	1.8 [0.8–3.9]	1.0 [0.3–3.0]	5.0 [2.0–9.0]
eGFR (mL/min/1.73 m^2^)	68 [56–81]	85 [71–96]	85 [70–95]	80 [66–90]	72 [57–89]	75 [61–87]	80 [65–92]	97 [87–104]
Total cholesterol (mmol/L)	4.5 [3.9–5.1]	3.9 [3.3–4.7]	5.0 [4.3–6.0]	4.5 [3.8–5.5]	3.9 [3.3–4.6]	3.9 [3.4–4.4]	3.7 [3.2–4.2]	3.7 [3.2–4.3]
HDL cholesterol (mmol/L)	1.3 [1.1–1.6]	1.2 [1.0–1.4]	1.2 [1.0–1.5]	1.3 [1.0–1.5]	1.1 [0.9–1.3]	1.4 [1.1–1.8]	1.0 [0.9–1.2]	1.2 [1.0–1.4]
LDL cholesterol (mmol/L)	-	-	3.4 [2.7–4.2]	2.8 [2.2–3.7]	1.9 [1.5–2.5]	2.0 [1.6–2.5]	2.0 [1.7–2.4]	1.9 [1.5–2.3]
Triglycerides (mmol/L)	1.3 [1.0–1.9]	1.5 [1.1–2.1]	1.6 [1.1–2.2]	1.5 [1.0–2.1]	-	0.9 [0.7–1.3]	1.1 [0.9–1.6]	1.3 [0.9–1.8]
Diabetes mellitus	19 (28 476)	25 (236 590)	14 (786)	16 (942)	17 (185)	20 (131)	24 (231)	23 (20 329)
History of coronary artery disease	70 (102 958)	70 (651 035)	66 (3739)	62 (3746)	100 (1112)	18 (118)	100 (946)	100 (87 847)
History of cerebrovascular disease	30 (43 609)	25 (232 250)	29 (1643)	23 (1412)	7.1 (79)	100 (654)	2.7 (26)	4.2 (3714)
History of peripheral artery disease	15 (21 950)	5.5 (51 605)	22 (1262)	24 (1441)	9.0 (100)	8.3 (54)	3.8 (36)	4.1 (3566)
History of abdominal aortic aneurysm	2.7 (4009)	4.9 (45 590)	-	-	-	-	0.4 (4)	1.3 (1118)
Years since first atherosclerotic CV disease diagnosis	-	7 [3–13]	-	-	**-**	**-**	1 [1–1]	0 [0–0]
Use of antithrombotics	87 (128 534)	89 (833 445)	s	-	99 (1101)	98 (643)	95 (900)	97 (85 588)
Follow-up (years)	6.1 [3.1–9.0]	2.3 [2.3–2.3]	12.0 [5.8–13.8]	1.8 [1.3–2.1]	7.0 [6.8–7.5]	2.2 [1.8–2.6]	5.0 [2.4–6.9]	4.7 [1.9–8.2]
Rate of recurrent CV events/1000 PY* [95% CI]	30 [30–30]	26 [26–26]	37 [35–38]	26 [23–29]	29 [25–34]	60 [48–75]	24 [19–29]	31 [30–31]
Rate of non-CV death/1000 PY* [95% CI]	27 [27–28]	16 [15–16]	20 [19–22]	12 [10–15]	6 [4–8]	29 [21–40]	7 [5–10]	13 [12–13]

	Karelia	Bialystok P/P	Estonian Biobank	SURF Serbia	EUROASPIREV	REACH- EU	REACH non-EU	Veterans Affairs
Country	Finland	Poland	Estonia	Serbia	-	-	-	US
European risk region	Moderate risk	High risk	High risk	Very High risk	Low-very high	Low-very high	-	-
Number of patients	3303	243	11 669	203	7044	18 100	28 407	930 725
Age (years)	70 ± 9	65 ± 9	65 [57–72]	65 ± 9	64 ± 9	65 ± 9	68 ± 9	73 ± 10
Male sex	69 (2289)	75 (182)	42 (4909)	70 (143)	74 (5232)	72 (13 046)	67 (19 028)	98 (912 063)
Current smoking	22 (732)	20 (48)	15 (1768)	30 (61)	16 (1135)	18 (3307)	14 (4020)	44 (404 454)
Systolic blood pressure (mmHg)	141 [129–158]	131 [121–146]	135 [120–145]	130 [120–140]	132 [121–145]	140 [130–152]	130 [120–145]	132 [120–142]
BMI (kg/m^2^)	28 [25–31]	29 [27–33]	28 [25–32]	27 [25–29]	29 [26–32]	27 [25–30]	27 [24–31]	-
C-reactive protein (mg/L)	1.8 [1.8–2.2]	1.1 [0.5–2.5]	2.4 [1.1–5.1]	0.8 [0.5–1.8]	-	2.1 [1.0–4.6]	2.1 [1.0–4.7]	-
eGFR (mL/min/1.73 m^2^)	79 [63–90]	77 [65–90]	77 [59–92]	83 [67–92]	79 [64–91]	73 [59–87]	71 [56–87]	70 [54–83]
Total cholesterol (mmol/L)	4.1 [3.3–5.1]	4.0 [3.3–4.7]	5.5 [4.7–6.3]	4.4 [3.6–5.2]	4.0 [3.4–4.8]	5.2 [4.4–6.0]	4.6 [4.0–5.4]	-
HDL cholesterol (mmol/L)	1.2 [1.0–1.5]	1.2 [1.1–1.6]	1.3 [1.0–1.7]	1.1 [0.9–1.2]	1.1 [0.9–1.3]	1.2 [1.0–1.5]	1.2 [1.0–1.4]	-
LDL cholesterol (mmol/L)	2.5 [1.8–3.3]	2.0 [1.5–2.5]	3.0 [2.4–3.9]	2.7 [2.1–3.5]	2.1 [1.7–2.8]	3.1 [2.5–3.8]	2.7 [2.1–3.3]	-
Triglycerides (mmol/L)	1.3 [0.9–1.8]	1.2 [0.8–1.8]	-	1.1 [0.9–1.4]	1.4 [1.0–1.9]	1.6 [1.1–2.2]	1.5 [1.1–2.2]	-
Diabetes mellitus	30 (1004)	31 (76)	18 (2122)	34 (70)	30 (2098)	32 (5749)	42 (11 955)	51 (470 804)
History of coronary artery disease	91 (3016)	74 (181)	91 (10 571)	100 (203)	100 (7044)	71 (12 871)	73 (20 856)	81 (751 706)
History of cerebrovascular disease	6.8 (225)	7.0 (17)	9.9 (1151)	3.4 (7)	4.3 (306)	33 (5951)	33 (9385)	**-**
History of peripheral artery disease	8.1 (269)	9.1 (22)	9.8 (1146)	2.0 (4)	2.9 (202)	20 (3681)	11 (3149)	39 (362 064)
History of abdominal aortic aneurysm	1.4 (47)	-	0.6 (66)	3.0 (6)	-	3.0 (550)	3.3 (930)	1.4 (13 316)
Years since first atherosclerotic CV disease diagnosis	0 [0–2]	2 [1–4]	5 [2–10]	0 [0–2]	2 [1–8]	1 [0–11]	1 [0–10]	6 [2–12]
Use of antithrombotics	-	96 (233)	-	96 (195)	96 (6776)	70 (12 646)	73 (20 831)	-
Follow-up (years)	2.2 [1.0–3.5]	2.8 [2.4–3.4]	2.0 [0.4–9.1]	3.9 [3.2–5.0]	1.3 [1.1–1.6]	1.8 [1.6–1.9]	1.8 [1.6–1.9]	3.5 [2.1–3.8]
Rate of recurrent CV events/1000 PY* [95% CI]	43 [39–48]	37 [24–56]	64 [62–67]	59 [44–79]	26 [23–30]	63 [60–66]	50 [48–52]	100 [100–100]
Rate of non-CV death/1000 PY* [95% CI]	20 [17–24]	17 [9–31]	10 [9–11]	3 [0–9]	4 [2–5]	13 [12–15]	14 [13–15]	23 [23–23]

Data are presented as % (*n*), mean ± standard deviation, median [25th–75th percentile] as appropriate.

SBP, systolic blood pressure: BMI, body mass index; CI, confidence interval; CRP, C-reactive protein; eGFR, estimated glomerular filtration rate; HDL, high-density lipoprotein; LDL, low-density lipoprotein; CV, cardiovascular; PY, person-years; EU, Europe.

The event rate was estimated based on the total follow-up time and all events in the cohort adjusted for competing risks (i.e. the event rate for non-CV death may be higher; however, the model is adjusted for competing risks.

The regional recalibration factors incorporated onto the base model are available in Supplementary data online, *Table S9*. The final model showed adequate calibration across risk regions for both endpoints (*Figures 1–3* and Supplementary data online, *Figures S6–8*). Age-specific calibration plots showed good agreements between the average predicted risk and observed incidences across all ages from 40 and older (see Supplementary data online, *Figures S9–11*). In additional analysis, good agreement between predicted and observed risks in those eligible only for healthcare through the VA was demonstrated, suggesting the miscalibration to be the result of events registered outside of the VA (see Supplementary data online, *Figure S12*). The pooled C-statistic across all cohorts for the risk of recurrent CV events was 0.68 [95% confidence interval 0.66–0.69] (*Figure 4*). The C-statistics for recurrent CV events within the four European risk regions were 0.66 [0.64–0.69] for the low-risk region, 0.69 [0.67–0.70] for the moderate-risk region, 0.70 [0.64–0.75] for the high-risk region, and 0.68 [0.63–0.73] for the very-high-risk region (see Supplementary data online, *Figures S13–15*). For non-European regions, the pooled C-statistic was 0.62 [0.56–0.69] and ranged from 0.62 [0.62–0.62] for the Veterans Affairs cohort to 0.72 [0.66–0.78] for Latin America (see Supplementary data online, *Figure S16*). The C-statistics for non-CV death were 0.73 [0.68–0.78] for the low-risk region, 0.69 [0.65–0.73] for the moderate-risk region, 0.68 [0.65–0.72] for the high-risk region and 0.66 [0.62–0.70] for the very-high-risk region, with a pooled estimate across all cohorts of 0.69 [0.66–0.71] (see Supplementary data online, *Figure S17*). Calibration and C-statistics for both endpoints were comparable in subgroups of sex (see Supplementary data online, *Figures S18*–*23*). Comparison with the original SMART-REACH model demonstrated comparable calibration and clinical utility in the low-risk region. However, in the moderate-risk region, SMART-REACH2 maintained adequate calibration whilst the original model underestimated risk, resulting in higher clinical utility for the updated model (see Supplementary data online, *Figure S24*).

**Figure 1:**
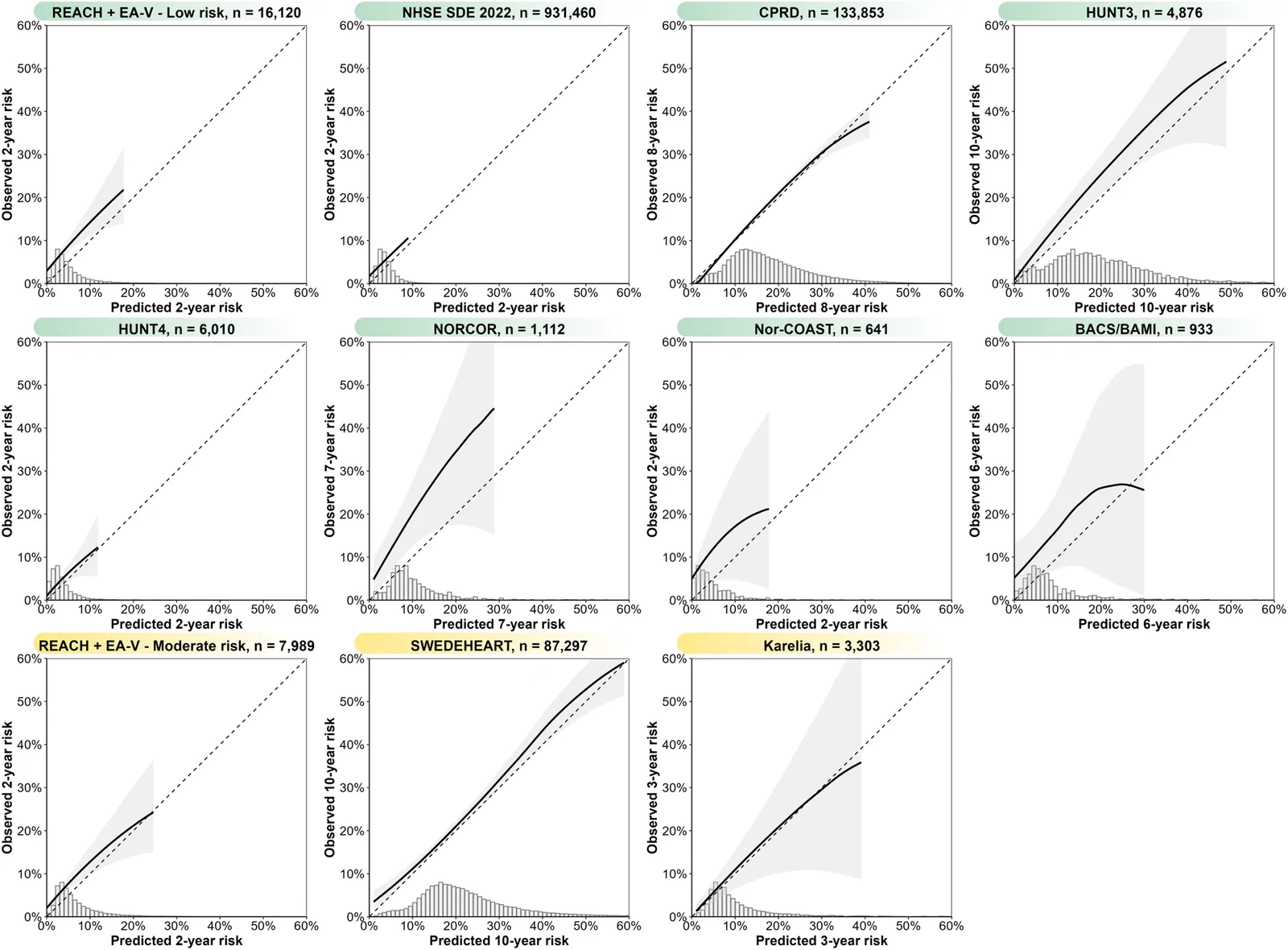
Calibration plots display predicted risk (*x*-axis) against observed risk (*y*-axis), with 95% confidence intervals. Histograms show the distribution of predicted risks. Cohort sizes here may be lower than the total cohort population listed in *Table 2* as risk can only be estimated up to age 90 (e.g. 89-year-olds can have 1-year risk but no risks for 2-year or higher and are consequently dropped at the horizon for validation). Cohorts belonging to the low-risk region are highlighted in green and cohorts belonging to the moderate region are highlighted in yellow. EA-V, EUROASPIRE V

**Figure 2:**
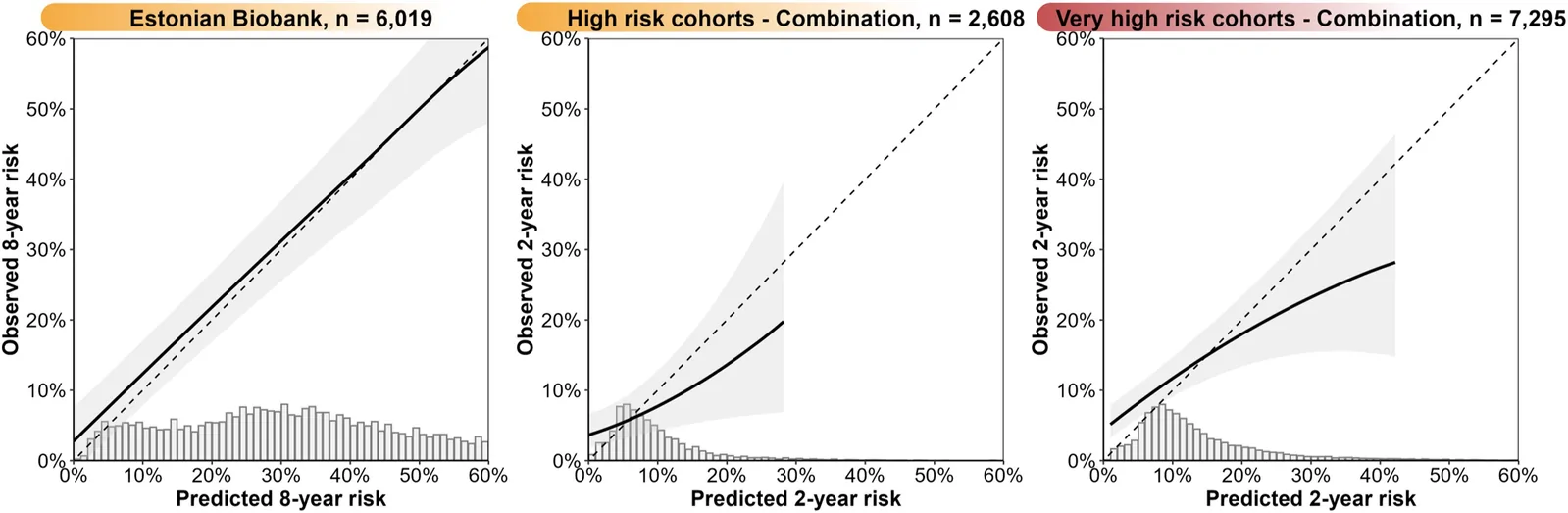
Calibration plots display predicted risk (*x*-axis) against observed risk (*y*-axis), with 95% confidence intervals. Histograms show the distribution of predicted risks. Cohort sizes here may be lower than the total cohort population listed in *Table 2* as risk can only be estimated up to age 90 (e.g. 89-year-olds can have 1-year risk but no risks for 2 years or higher and are consequently dropped at the horizon for validation). Cohorts belonging to the high-risk region are highlighted in orange and cohorts belonging to the very-high-risk region are highlighted in red. The combination of high-risk cohorts consists of REACH, EUROASPIRE-V and Bialystok POLASPIRE, the combination for the very-high-risk region consists of REACH, EUROASPIRE and SURF-CHD

**Figure 3:**
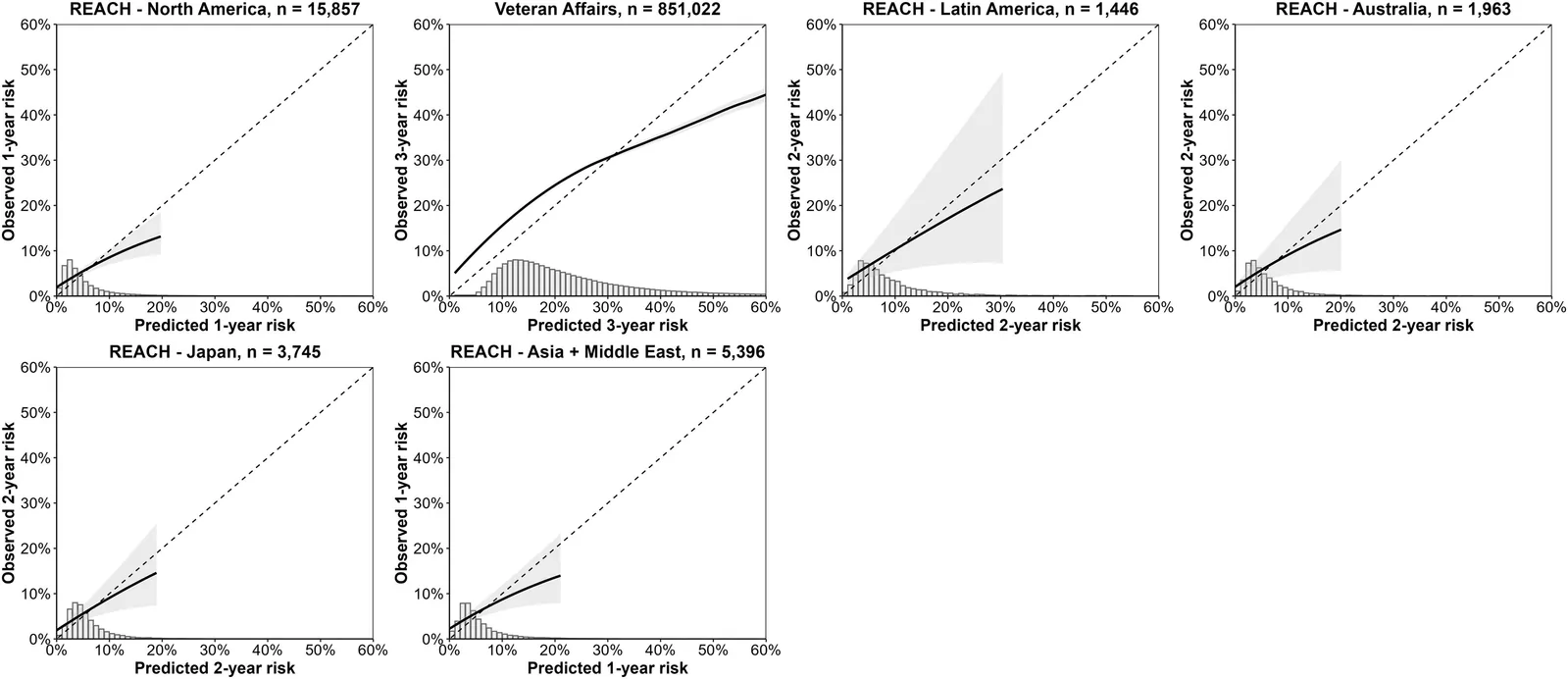
Calibration plots display predicted risk (*x*-axis) against observed risk (*y*-axis), with 95% confidence intervals. Histograms show the distribution of predicted risks. Cohort sizes here may be lower than the total cohort population listed in *Table 2* as risk can only be estimated up to age 90 (e.g. 89-year-olds can have 1-year risk but no risks for 2 years or higher and are consequently dropped at the horizon for validation). Classification according to European risk regions do not apply and as such are not highlighted in the title

**Figure 4:**
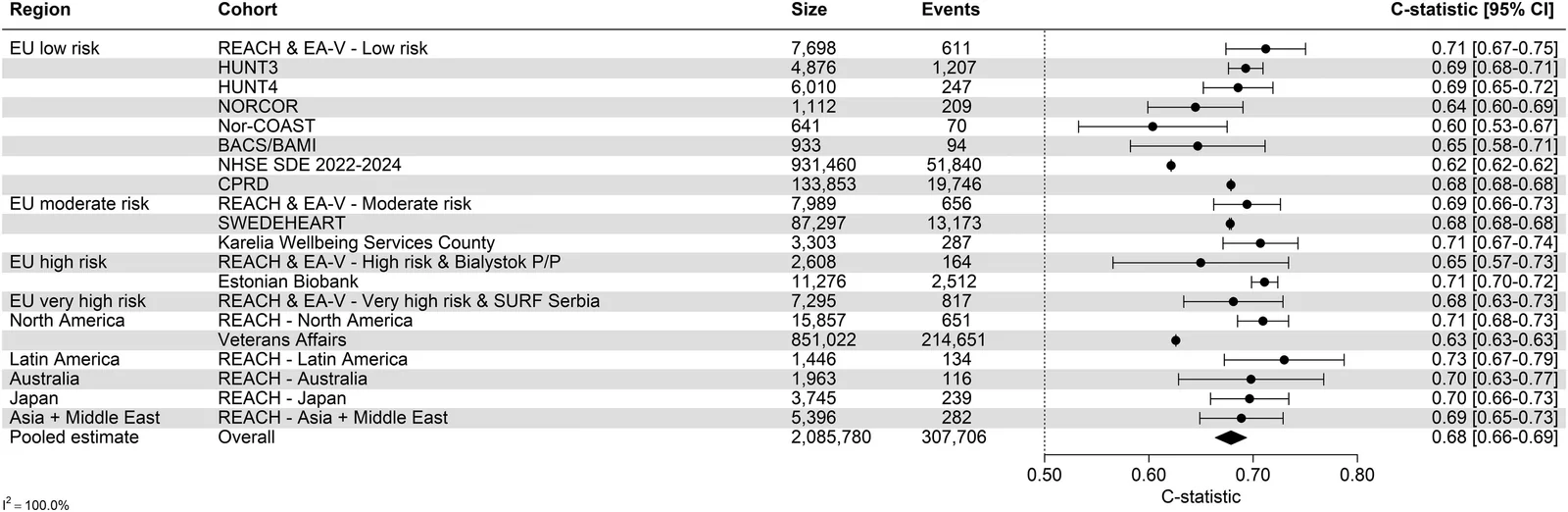
The forest plot displays the pooled discrimination as measured by C-statistic for recurrent CV events adjusted for the competing endpoint (non-CV death). Cohort sizes here may be lower than the total cohort population listed in *Table 2* as risk can only be estimated up to age 90 (e.g. 89-year-olds can have 1-year risk but no risks for 2 years or higher and are consequently dropped at the horizon for validation). CV, cardiovascular; CI, confidence interval; EU, Europe; EA-V, EUROASPIRE -V; I^2^, heterogeneity

### Sensitivity analyses

Discrimination for both recurrent CV events and non-CV death was similar across specific CV disease subtypes (see Supplementary data online, *Figures S25* and *S26*). Restricting the analyses to patients with a single CV disease subtype did not change the results. Adequate calibration was observed across different CV subtypes (see Supplementary data online, *Figure S27*). Model performance in the NHSE SDE COVID-19 pandemic-specific cohort showed underprediction for non-CV death and mild underprediction for recurrent CV events (see Supplementary data online, *Figure S28*). This analysis was performed according to a pre-specified analysis plan published on GitHub (https://umcutrecht-my.sharepoint.com/personal/j_holtrop-3_umcutrecht_nl/Documents/Documenten/GitHub), along with the phenotyping definitions, and data curation and analysis code.

### Example of estimation of treatment effects

A clinical example of the possible use of SMART-REACH2 for estimation of long-term gain from SBP and LDL cholesterol lowering is available in *Figure 5*. The modelled gain in CV disease-free life expectancy reflects for the combined effect of lowering SBP by 15 mmHg and LDL cholesterol by 1.0 mmol/L for the same clinical vignette for each of the four European risk regions. These example patients are males aged 50 with a history of CAD for 1 year with the following risk factor levels: SBP 150 mmHg, CRP 2 mg/L, eGFR 75 mL/min/1.73 m^2^, non-HDL cholesterol of 2.8 mmol/L with an LDL cholesterol of 1.8 mmol/L. The gain in CV disease-free life expectancy for this intensification of preventive treatment was 2.0 years for the patient from the low-risk region, 3.1 for the moderate-risk region, 4.0 years for the high-risk region, and 4.4 years for the very-high-risk region.

**Figure 5:**
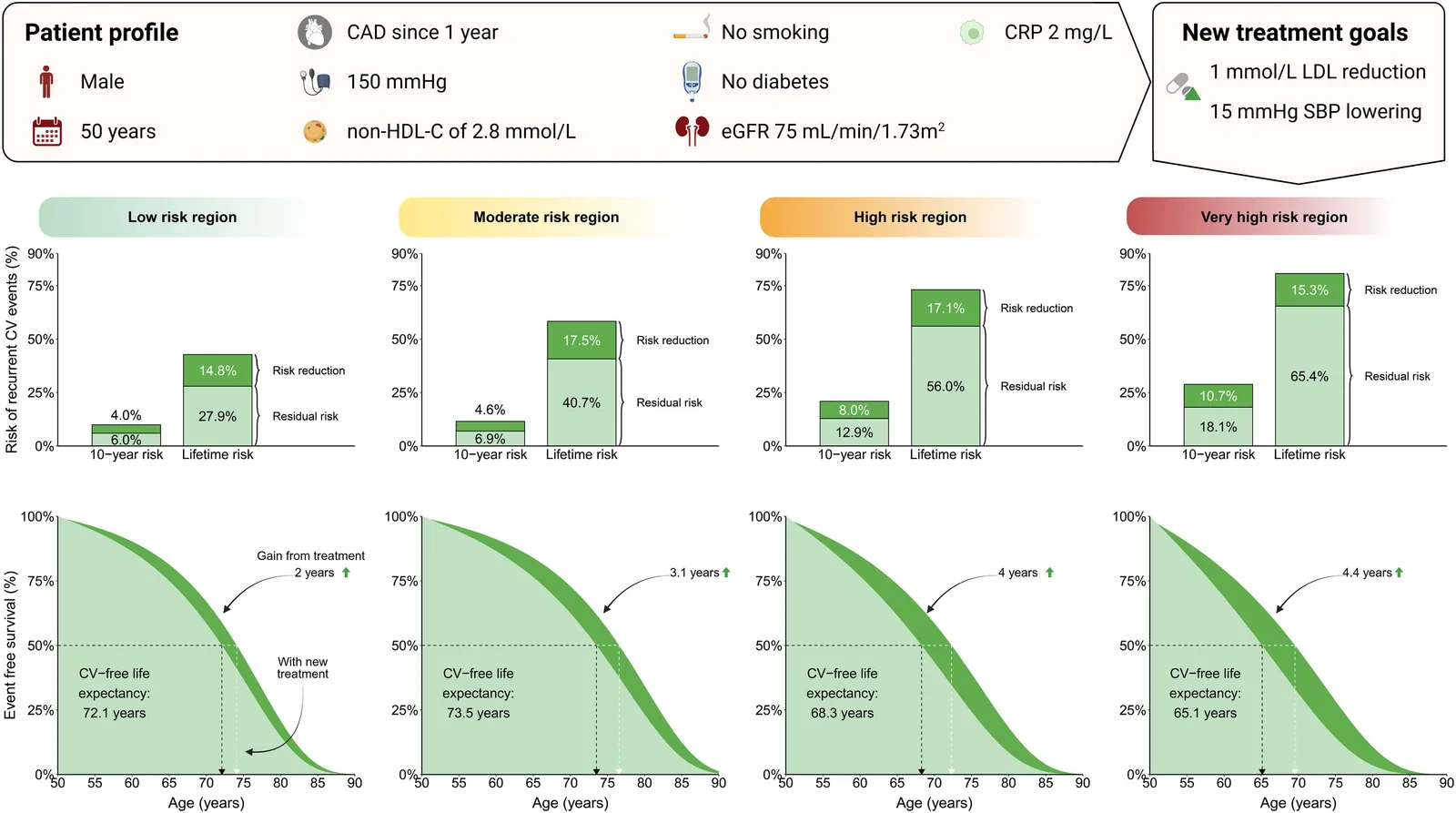
Estimation of cardiovascular disease-free life expectancy for example patients. CAD, coronary artery disease; HDL-C, high-density lipoprotein cholesterol; eGFR, estimated glomerular filtration rate; CRP, C-reactive protein; LDL, low-density lipoprotein; SBP, systolic blood pressure. CV, cardiovascular. Created with BioRender.com

## Discussion

The current study describes the development of the SMART-REACH2 model for patients with established atherosclerotic CV disease, its geographical recalibration, and external validation in over 2 million patients. The SMART-REACH2 model can be used in all four of the European and other global risk regions to estimate 10-year as well as lifetime risk of recurrent CV events, CV disease-free life expectancy, and potential benefits of preventive treatment on an individual level (*Structured Graphical Abstract*).

The 2021 ESC Guidelines on CV disease prevention in clinical practice recommend using short- and long-term risk models to support treatment decisions.^2^ Recommended short-term prediction models for secondary prevention include EUROASPIRE for the 2-year risk in patients with CAD and SMART2 for the 10-year risk in patients with atherosclerotic CV disease. External validation of these models in SWEDEHEART reported C-statistics between 0.64 and 0.69, similar to SMART-REACH2 in this cohort.^37,38^ These are typical of prediction models for secondary prevention populations, where the inherently high risk of events naturally limits the maximum achievable C-statistic.^39^ Short-term prediction models tend to identify older patients as being at high risk, as age is a major driver of CV risk.^3^ Lifetime risk models, such as SMART-REACH2, could potentially complement short-term approaches by identifying younger high-risk individuals, enabling early intervention and more long-term preventive strategies. To allow for synergistic use SMART2 and SMART-REACH2 use identical predictors and apply to the same patients. Importantly both models are recalibrated to the same European and other global risk regions. This alignment allows for consistent risk communication and shared decision-making while reducing implementation barriers, such as integration into clinical workflows. Lifetime risk for the support of treatment decisions may be cost-effective, inform policy makers, physicians, and patients.^4,5^ Apart from informing shared decision-making on treatment decisions risk estimates could positively impact patients’ health behaviour thus serving a double purpose.^5^ SMART-REACH2 offers an additional advantage by providing estimates of CV disease-free life expectancy. Such estimates may increase patient comprehension as risk numerical estimates (i.e. percentages) may be difficult to understand even for more highly educated patients.^6^

The SMART-REACH2 model builds on the original SMART-REACH model in several ways. First, the minimum age of patients for whom the model can be used was lowered from 45 to 40 years. Although a modest change, it allows for identification of patients at high risk at an even younger age. As these youngers patients are generally those with potentially higher lifetime treatment benefit, this could aid in more effective allocation of preventive treatment on both an individual and population level.^4,20^ Second, the models were derived sex-specifically. This is especially relevant as sex-specific variations between risk factors and CV disease risk, such as diabetes mellitus, have been shown to exist in prior studies.^40^ Ignoring these differences could lead to mis-specified predictor associations for either sex potentially leading to inaccurate risk estimation and suboptimal treatment decisions.^8^ Third, the SMART-REACH2 model is recalibrated to the four European risk regions. Accounting for more of the geographical variation in CV disease background risk reduces the potential for over- or underestimation of risk. This ensures that patients in different risk regions receive accurate risk predictions, improving the model's applicability for clinicians across varied healthcare settings. Fourth, the SMART-REACH2 model utilizes more contemporary data, thus ensuring that model predictions are better suited for the patients of today. Importantly, this data includes routine clinical care data which, by its nature, represents those patients seen in everyday clinical practice. Through recalibration using several large and representative datasets, performance across all risk regions was overall adequate. However, further validation and recalibration efforts would strengthen the model in several contexts. Whilst these datasets include more data from the European high- and very-high-risk regions and North America than used before, additional external validation in these regions would further strengthen applicability. Model performance should be monitored over time to account for temporal changes in cardiovascular disease incidence, risk factor profiles, and treatment patterns. Additionally, targeted validation in ethnic minorities and those from lower socioeconomic backgrounds should be considered as risk dynamics in these subgroups vary substantially between countries. Where needed, local recalibration can account for such population-specific dynamics as well as differences in healthcare systems and coding practices. To facilitate these efforts, reproducible code and the theoretical framework for recalibration are provided. The SMART-REACH2 model uses predictors available across all lines of care, enabling broad applicability. Various factors may refine risk estimation beyond these predictors, such as lipoprotein(a) and urinary albumin-to-creatinine ratio.^2^ However, such factors are not routinely measured and may restrict the model's applicability. Future extensions could integrate these predictors when available using flexible add-on approaches.^41^ Nevertheless, discrimination improvements from additional predictors are inherently limited in established models. For instance, adding biomarkers or multi-protein scores to existing models yields ΔC-statistics of 0.01–0.04, reflecting natural limits based on baseline model performance and predictor characteristics.^41,42,43^

Strengths of the current study include, firstly, the derivation in a prospective cohort study with that includes all of the combinations of atherosclerotic CV disease subtypes, systematic baseline assessment, and long follow-up. Secondly, the extensive external validation in over 2 million patients and geographical recalibration, including electronic health record data, which ensures wide applicability of SMART-REACH2. Thirdly, the use of routinely available predictor information enables implementation in all regions as well as healthcare setting, thus providing consistency for patients and health care providers. Lastly, compared to more short-term models, SMART-REACH2’ lifetime risk predictions potentially allow for easier patient communication and promote shared decision making. Limitations of the current study have to be considered. First, the model was developed in a single-centre cohort from a low-risk region, selected for its long-term follow-up, systematic data collection, minimal missing data, and adjudicated outcomes. To address generalizability, extensive external validation and recalibration were performed in over 2 million patients from multiple large datasets across diverse risk regions, demonstrating adequate performance. Second, validation of lifetime risk is not practically feasible, as obtaining lifetime yet contemporary follow-up data is impossible.^3^ However, the methods used for SMART-REACH2 have shown to yield accurate predictions at least up to 17 years of follow-up.^31^ The age-specific calibration plots demonstrate adequate agreement between predicted and observed risk across the age range to which SMART-REACH2 applies. This agreement supports the lifetime risks estimated by the SMART-REACH2 model. Nevertheless, in the absence of full lifetime validation, SMART-REACH2 should not be interpreted as providing empirically observed lifetime outcomes. Rather, it is a tool to estimate long-term risk and potential treatment benefit based on validated methods that account for competing risks and age-related hazard patterns. Third, most validation data are from the low and moderate European risk regions. High quality datasets for secondary prevention are scarcer in the high- and very-high-risk as well as other global regions. Ideally, the model would be recalibrated to larger population-based registries in these regions as well. Nonetheless the model was recalibrated using the best available data.

In conclusion, the updated SMART-REACH2 model accounts for geographical and sex-specific variations and allows estimation of short-term and lifetime risk of recurrent CV event risk and treatment benefits across Europe and other global risk regions, facilitating shared decision-making as recommended by guidelines.

## Supplementary Material

ehag400_Supplementary_Data

## Declarations

### Data Availability

The data used for derivation of the SMART-REACH2 model can be applied for here (https://www.umcutrecht.nl/en/ucc-smart). The North East-Newcastle and North Tyneside 2 research ethics committee provided ethical approval for the CVD-COVID-UK/COVID-IMPACT research programme (REC No 20/NE/0161) to access, within secure trusted research environments, unconsented, whole-population, anonymized data from electronic health records collected as part of patients’ routine healthcare.

The data used in this study are available in NHS England's Secure Data Environment (SDE) service for England, but as restrictions apply they are not publicly available (https://digital.nhs.uk/services/secure-data-environment-service). The CVD-COVID-UK/COVID-IMPACT research programme, led by the BHF Data Science Centre (https://bhfdatasciencecentre.org/), received approval to access data in NHS England's SDE service for England from the Advisory Group for Data (AGD) (https://digital.nhs.uk/about-nhs-digital/corporate-information-and-documents/advisory-group-for-data)—formerly the Independent Group Advising on the Release of Data (IGARD)—via an application made in the Data Access Request Service (DARS) Online system (ref. DARS-NIC-381078-Y9C5K) (https://digital.nhs.uk/services/data-access-request-service-dars/dars-products-and-services). The CVD-COVID-UK/COVID-IMPACT Approvals & Oversight Board (https://bhfdatasciencecentre.org/areas/cvd-covid-uk-covid-impact/) subsequently granted approval to this project (CCU004) to access the data within NHS England's SDE service for England. The anonymized data used in this study were made available to accredited researchers only. Those wishing to gain access to the data should follow the application process of the relevant national data custodian.
