# Supplementary material for: Predicting lifetime cardiovascular risk and benefits of preventive treatment in patients with established atherosclerotic cardiovascular disease: the SMART-REACH2 model
Source: Eur Heart J. 2026 Jun 5;47(39):5840–56. doi: 10.1093/eurheartj/ehag400 (PMC13690821; doi:10.1093/eurheartj/ehag400)
Supplement: Supplementary file 1 [file ehag400_supplementary_data.docx]

**Supplementary Material**

**Table of contents**

Use Ctrl + mouse click to navigate directly to the table mentioned in the Table of contents

**Supplementary Methods**

Methods S1. External validation cohorts

Methods S2. Shrinkage

Methods S3. Estimation of baseline hazards

Methods S4. Missing data

Methods S5. Lifetable estimation

Methods S6. Calibration plots

Methods S7. Recalibration

Methods S8. Estimation of treatment benefits

**Supplementary Tables**

Supplementary Table 1. Definitions of atherosclerotic CV disease at baseline in UCC-SMART

Supplementary Table 2. Definition of history of vascular disease or diabetes mellitus at baseline

Supplementary Table 3. Endpoint definitions

Supplementary Table 4. Proportion of missing data in predictor variables and outcomes in UCC-SMART cohort

Supplementary Table 5. Penalized sex-specific hazard ratios

Supplementary Table 6. Model coefficients for respective endpoints per sex

Supplementary Table 7. Sex-specific means and standard deviations used for scaling and centring

Supplementary Table 8. Interval specific baseline survival for respective endpoints per sex

Supplementary Table 9. Expected observed ratios for recalibration to the respective risk regions.

**Supplementary Figures**

Supplementary Figure 1. Origin of European data sources in relation to the four European risk regions

Supplementary Figure 2. Steps for risk estimation using SMART-REACH2

Supplementary Figure 3. Baseline hazards for recurrent CV events and non-CV death in women

Supplementary Figure 4. Baseline hazards for recurrent CV events and non-CV death in men

Supplementary Figure 5. Internal validation in UCC-SMART

Supplementary Figure 6. Calibration for non-CV death in external validation cohorts from the European low and moderate-risk regions

Supplementary Figure 7. Calibration for non-CV death in external validation cohorts from European high- and very-high-risk regions

Supplementary Figure 8. Calibration for non-CV death in external validation cohorts outside of Europe

Supplementary Figure 9. Age-specific calibration for recurrent CV events in external validation cohorts from the European low- and moderate-risk regions

Supplementary Figure 10. Age-specific calibration for recurrent CV events in external validation cohorts from the European high- and very-high-risk regions

Supplementary Figure 11. Age-specific calibration for recurrent CV events in external validation cohorts outside of Europe

Supplementary Figure 12. External validation in the Veterans Affairs cohort for patients eligible only for health care through the Veterans Affairs

Supplementary Figure 13. Discrimination for recurrent CV events following regional recalibration for the European low-risk region

Supplementary Figure 14. Discrimination for recurrent CV events following regional recalibration for the European moderate-risk region

Supplementary Figure 15. Discrimination for recurrent CV events following regional recalibration for the European high- and very-high-risk region

Supplementary Figure 16. Discrimination for recurrent CV events following recalibration for the non-European risk regions

Supplementary Figure 17. Discrimination for non-CV death following regional recalibration

Supplementary Figure 18. Discrimination for recurrent CV events following regional recalibration according to sex

Supplementary Figure 19. Discrimination for non-CV death following regional recalibration according to sex

Supplementary Figure 20. Calibration for recurrent CV events in women following regional recalibration

Supplementary Figure 21. Calibration for recurrent CV events in men following regional recalibration

Supplementary Figure 22. Calibration for non-CV death in men following regional recalibration

Supplementary Figure 23. Calibration for non-CV death in women following regional recalibration

Supplementary Figure 24. Comparison of the SMART-REACH and SMART-REACH2 model

Supplementary Figure 25. Discrimination for recurrent CV events according CV disease subtypes following regional recalibration

Supplementary Figure 26. Discrimination for non-CV death according CV disease subtypes following regional recalibration

Supplementary Figure 27. Calibration of recurrent CV events according to CV disease subtypes in HUNT3

Supplementary Figure 28. Calibration for recurrent CV events and non-CV death in the NHSE SDE cohort during the COVID-19 pandemic

# Supplementary methods

## Methods S1. External validation cohorts

| Cohort |  |
| --- | --- |
| CPRD | The UK’s CPRD GOLD database is a repository for longitudinal individual primary care patient data collected from 1987 onwards. Over time, 966 primary care centres have contributed data for >20 million patients, with 407 practices actively contributing data for >3 million patients currently. For the most part CV disease prevention is undertaken in primary care and general practitioners are incentivised to use standard coding procedures to record this activity. For the current study, individuals with a history of CV disease between January 1st 2006-2008 were included. |
| NHSE SDE^13,43–45^ | The NHSE SDE data resource includes linked electronic health records from primary care (General Practice data for Pandemic Planning and Research, GDPPR), secondary care (Hospital Episode Statistics Admitted Patient Care), NHS (NHS Business Services Administration (NHS-BSA) Medicines Dispensed in Primary Care (MDPC)), and deaths (Office of National Statistics death registrations) covering >96% of the English population and 98% of all general practices. Data were accessed via the British Heart Foundation Data Science Centre’s [CVD-COVID-UK/COVID-IMPACT Consortium](https://eur05.safelinks.protection.outlook.com/?url=https%3A%2F%2Fbhfdatasciencecentre.org%2Fareas%2Fcvd-covid-uk-covid-impact%2F&data=05%7C02%7CJ.Holtrop-3%40umcutrecht.nl%7C9c54da7766fe43618dee08dd8f278826%7Cdcdf4a3dd0c04a6394cf781981249be5%7C0%7C0%7C638824122161450834%7CUnknown%7CTWFpbGZsb3d8eyJFbXB0eU1hcGkiOnRydWUsIlYiOiIwLjAuMDAwMCIsIlAiOiJXaW4zMiIsIkFOIjoiTWFpbCIsIldUIjoyfQ%3D%3D%7C0%7C%7C%7C&sdata=bAKua3K7QWCx7zFsPa2CMwuCfF%2Bphqg4nP79twjIJJE%3D&reserved=0) and is available for COVID-related research questions. Our aim was to quantify the performance of the SMART-REACH2 risk models during the pandemic period (2020-2022). and the post-pandemic period (2022-2024); in particular we hypothesised that the SMART-REACH2 model may underestimate risk of CVD and non-CVD endpoints during the pandemic period due to the direct and indirect impacts of COVID-19 on cardiovascular disease (3, 4) , but likely to perform better in the post-pandemic period. Two cohorts were created, the first spanning the COVID-19 period, from January 1^st^ 2020 to March 1^st^ 2022, and the second being a non-COVID cohort from March 2^nd^ 2022 to December 2024. Disease history was defined based on ICD-10 codes and only those patients with an atherosclerotic CV disease diagnosis at the start of the respective cohorts were included. Baseline measurements were selected as those being the closest to the start of the cohort and measured between 2-year prior up 6-months after start of the cohort. For the current analysis only, those events registered at secondary care were included as event. This analysis was performed according to a pre-specified analysis plan with the code lists to define variables and analysis code published on GitHub: <https://github.com/BHFDSC/CCU004_06>. |
| HUNT3 | HUNT3 is population-based health study in the Nord-Trøndelag County in Norway conducted between 2006-2008. Of the 93,860 eligible residents, all were contacted by post, and 50,807 participated (54.1%). Information on history of atherosclerotic CV disease was obtained via baseline questionnaires and linkage to the local hospital registry. Follow-up information was obtained by linkage to local and national registries for cardiovascular disease and mortality. |
| HUNT4 | HUNT4 is population-based health study in the Nord-Trøndelag County in Norway conducted between 2017-2019, the survey data from Sør-Trøndelag was not part of the current study. Of the 103,800 invitees from Nord-Trøndelag 56,042 (54.0%) participated. In total 33,819 participated both in HUNT3 and HUNT4. Information on history of atherosclerotic CV disease was obtained via baseline questionnaires and linkage to the local hospital registry. Follow-up information was obtained by linkage to local and national registries for cardiovascular disease and mortality. |
| Nor-COAST | Nor-COAST is a multicentre (5 centres, all in Norway), prospective, cohort study, consecutively including patients admitted with acute stroke between 2015 and 2017. Patients have follow-up visits at 3 and 18 months, and at 3 years. Patients with an expected survival of <3 month at the time of screening for eligibility were not included. For the current analysis, patients with ischemic stroke who were alive at the 3-month follow-up visit were included. Follow-up started at the 3-month follow-up visit. Follow-up information was obtained by linkage to national registries for cardiovascular disease and mortality. |
| NORCOR | NORCOR is a multicentre (2 hospitals in Norway), prospective cohort study including patients with a history of CAD (acute myocardial infarction (ICD-10; I.21), coronary artery by-pass graft operation (CABG), or elective or emergency percutaneous coronary intervention (PCI), defined as the index event/treatment) within the 3 years (2011-2014) prior to study inclusion. Patients with short life expectancy were excluded. Of all patients eligible for inclusion 83% agreed to participate. Follow-up information was obtained by linkage to national registries for cardiovascular disease and mortality. |
| BASC/BAMI | BACS/BAMI consisted of patients discharged from one of 5 hospitals in Madrid between July 2006 and June 2010 with CAD defined as non‐ST elevation acute coronary syndrome (ACS) or ST‐elevation myocardial infarction (MI). Patients with expected limited survival or impossibility of attending follow-up were excluded. Of those patients eligible 2% declined to participate. Follow-up for the current study started at this first follow-up visit, 6-12 months after inclusion events. |
| SWEDEHEART | SWEDEHEART is a nationwide quality registry that collects information on patient characteristics, in-hospital events, treatment and medications, at arrival, in-hospital, discharge, and follow-up visits from all Swedish coronary care units (n=72). For the current study, we included patients aged between 40-90 years old with a MI and who had a follow-up visit between 31-01-2005 and 01-03-2020. The follow-up visit at 6-10 weeks following discharge from the MI event was taken as baseline visit – follow-up started at this point. |
| Karelia Wellbeing service Country | The Karelia Wellbeing service Country cohort included patients aged 45-85 in North Karelia, Finland (population ~164,700) who experienced ACS or underwent coronary revascularization between January 2017 and December 2021. Data was collected from the region's unified electronic health record system, which covers 13 municipalities and captures approximately 600-700 new cases annually. Follow-up began either 7 days post-event or after hospital discharge (whichever was later) and continued through June 2022, ensuring a minimum 6-month follow-up period. Historical medical data was retrieved from January 2011 to the index event to establish patient comorbidities. |
| Estonian Biobank | The Estonian Biobank is a population-based study from the Estonian Genome Center at the University of Tartu. Patients from all 15 Estonian Counties participated recruiting patients from over 56% of all registered general practitioners. The total biobank covers around 5% (n = ~52, 000) of the Estonian population. From this all patients were included with established atherosclerotic CV disease prior to inclusion. All biobank participants have signed a broad informed consent form and the study was carried out under ethical approval 1.1-12/624 from the Estonian Committee on Bioethics and Human Research (Estonian Ministry of Social Affairs), using data according to release application 6-7/GI/10462 from the Estonian Biobank. |
| POLASPIRE | Bialystok PLUS/Polaspire included patients in 2016–2018 who were previously hospitalized for acute coronary event or elective percutaneous revascularisation procedure and were followed for a median time of 3 years. Patients were included in the study 12-18 months after the coronary event. Follow-up was performed through return visits at 1 and 3 years after baseline or by linkage to national mortality registers. |
| SURF CHD II Serbia | SURF CHD II – Serbia consists of the Serbian part of the cross-sectional SURF CHD II data performed between 2019 to 2022 in 105 centers located in 29 countries. Patients included were those with a previous diagnosis of CHD, including stable angina pectoris, ASC, PCI, and/or CABG. For the current study variables needed for risk estimation and not meased in SURF were extracted from electronic health records. As systematic follow-up is not part of SURF, this was obtained by local investigators by contacting next of kin. |
| EUROASPIRE V | EUROASPIRE V is a Europe wide investigation into guideline adherence in Europe conducted between 2016–2017 in 27 countries and 131 centres. Patients with an elective or emergency CABG, PCI, acute MI (ICD-10: I21) or acute myocardial ischaemia 6-24 months prior to inclusion were contacted consecutively. Of the 16,208 patients identified 8261 (56%) took part in the interview. Patients were followed prospectively for incident fatal and non-fatal cardiovascular events. For the current study patients with insufficient quality on endpoint status, defined as “unknown” status for two or more of the composite outcome of recurrent CV events, were excluded. |
| Veterans Affairs | The Department of Veterans Affairs (VA) in the US provides healthcare coverage to approximately 9 million US Veterans. The study was approved by the Institutional Review Board of the Northeast Ohio VA Healthcare system. One investigator (SVD) had access to the data and performed all the statistical analyses for the VA cohort. SVD solely is responsible for the validity of observations reported from the VA data. The data was housed within the VA informatics and computing infrastructure (VINCI) and only results were shared with the other investigators involved in this study. The cohort used for this analysis was derived from tables provided by the tables created by VINCI pooled from the electronic health record information.US Veterans that visited VA medical centers with a stable diagnosis of atherosclerotic CV disease ascertained by ICD-10 codes during January 2016 were included and the last date of follow-up was 31^st^ January 2020. Deaths during the follow-up period and the cause of death was obtained from the Mortality Data Repository (with approval); patients with missing information on vital status were excluded from the analysis. |
| REACH | In the international, prospective REACH Registry, participants were enrolled between 2003 and 2004 from physician outpatient practices in several global regions, including Western and Eastern Europe. REACH included patients with established CV disease (n = 53,211) or risk factors. The planned follow-up was planned for up to 2 years. Most sites contributed to the extended follow-up up to 4-years. Participants were followed for a maximum of 4 years for the occurrence of CV events and mortality. |
| CPRD, Clinical Practice Research Datalink; BHF, British Heart Foundation; HUNT, Norwegian Trøndelag Health Study; Nor-COAST, COgnitive Impairment After Stroke; NORCOR, NORwegian CORonary Prevention; BACS/BAMI, Spanish Biomarkers in Acute Coronary Syndrome and Biomarkers in Acute Myocardial Infarction; SWEDEHEART, Swedish Web‐System for Enhancement and Development of Evidence‐Based Care in Heart Disease Evaluated According to Recommended Therapies; POLASPIRE, SURF CHD II, Survey in Risk Factors in Coronary Heart Disease II; VA, Veterans Affairs; EUROASPIRE, European European Action on Secondary and Primary Prevention by Intervention to Reduce Events; REACH, REduction of Atherothrombosis for Continued Health; UK, United Kingdom; CV, cardiovascular disease; COVID, coronavirus disease; ICD, international classification of disease; CAD, coronary artery disease; PCI, percutaneous coronary intervention; CABG, coronary artery bypass graft; ACS, acute coronary syndrome, MI, myocardial infarction; US, united states | |

Methods S2. Shrinkage

Statistical prognostic models are optimized for the derivation data and often perform less well in external validation on independent dataset. Such differences in performance can arise from, among others, difference in definitions (in outcome or predictors), differences in case-mix (e.g., patients managed for their CV risk in primary care differ from those that were referred for to a cardiologist), and overfitting^46^. For the current study ridge regression with penalized likelihood estimation was performed to minimize the risk of overfitting (i.e., optimism correction). Additionally, the use of predefined predictors for SMART-REACH2 avoids the data driven selection and thus reduces risk of overfitting.

For the current analysis the optL2 function (Penalized package in R) was used to find the optimal value of lambda for shrinkage for both endpoints individually. Cross-validation using a 5-fold split was used with identical splits for both endpoints. No restrictions were set on the number of events per split as the number of events is likely to vary in external datasets. Continuous predictors were scaled and centred using the sex-specific means and standard deviations. Binary variables were not scaled. Use of antithrombotic medication was included as an (unpenalized) offset in the model for recurrent CV events.

The use of an offset term for antithrombotic medication serves a double purpose. First, use of medication is related to reasons for prescription influenced by local practice and changes in guidelines. As such, the use of medication is related to prognostic factor. For example, use if aspirin is indicated following a myocardial infarction, however for patient that has suffered from severe bleeding a different choice might be made regarding the use of medication. Consequently, estimating a coefficient for antithrombotic use from the data is related to all these patterns, which can differ from dataset to dataset due to differences in case-mix and health care setting. This not only leads to a less generalizable coefficient for antithrombotic use, but also influences factors that are related to it, such as the coefficient for coronary artery disease.

Methods S3. Estimation of baseline hazards

The use of age as timescale allows for estimation of age-specific conditional survival probabilities for specific time intervals from the fitted cause-specific Cox models. These estimates were calculated for 1-year intervals (e.g., the probability of turning 51 given that one has turned 50 years old) for the respective endpoints. The 1-year survival probabilities for ages 40-90 were obtained for men and women separately using a reference patient with all risk factors set to their sex-specific means and binary predictors set to 0.

Due to chance and limited sample size (especially in younger patients with established atherosclerotic CV disease), interval-specific survival probabilities can sometimes be estimated as 100% (i.e., no person experiencing an event regardless of risk factor levels). As these are unrealistic in real-world applications, an exponential model with an upper bound at the maximum survival probability was fitted. Smoothing of survival probabilities in this way leads to a more intuitive progression of baseline survival with age and avoids unrealistic survival probabilities.

The nls function from the stats package in R was used to fit the exponential decay model: S(t) = S_max_ - a * e^(-β * age)^, where S_max_ is the maximum survival probability functioning as upper bound, α is the amplitude parameter, and β controls the decay of the survival probability with age. S_max_ was set to the highest survival probability observed among patients aged between 40-45. The exponential model was fitted using the number of observations contributing to each age interval as weights to account for varying precision of estimates across age groups.

Methods S4. Missing data

Missing data for the derivation was addressed using multiple imputation with a total of 15 datasets. Estimates where pooled using Rubin’s rules where appropriate. To ensure congeniality both outcomes were included in the imputation using Nelson-Aalen estimators. The outcomes and follow-up time itself were complete. Additionally, the polynomials and transformations considered for the predictor variables were included as passive predictors in the imputation. The mice package in R was used for imputation using predictive mean matching for continuous variables and logistic regression for binary variables. Only predictor values were imputed, other values reported in the baseline table are unimputed. The best transformation was selected by using a median AIC across imputations. The scaling and centring was performed on each dataset using the pooled means and standard deviations.

*External validation*
Missing data was handled in each cohort separately and using different approaches for sporadically (i.e., data that was available for some patients) and systematically missing data (i.e., data that was available for none). For data that was sporadically missing the same approach as for the derivation was used regarding imputation using mice and inclusion of the outcomes. Datasets where larger portions of missingness relevant for recalibration used multiple imputation instead of single imputation. These include CPRD, NHSE SDE and REACH. Ideally, systematically missing variables were handled using multilevel multiple imputation with fully conditional specification via the mitml-impute package in R (5 imputed datasets). However, as this required the data being transferred to be combined with the other datasets, this was only possible for the REACH Registry (HDL-cholesterol, CRP, years since first atherosclerotic CV disease diagnosis). For CPRD and NHSE SDE a total of 5 imputed datasets were created using MICE (*mice* package in R). For those cohorts using multiple imputation results were pooled using Rubin’s rules (takin into account within- and between variance).

For systematically missing variables in other cohorts’ median imputation was used or different assumptions were made. The use of median imputation has been shown to yield results that are non-inferior to complex imputation methods for the external validation of prediction modelling^47^. Median imputation was used in HUNT3 (prevalence of AAA), NORCOR (prevalence of AAA and years since first atherosclerotic CV disease diagnosis), Nor-COAST (NORCOR (prevalence of AAA and years since first atherosclerotic disease diagnosis), EUROASPIRE-V (prevalence of AAA), POLASPIRE (prevalence of AAA) and the NHSE SDE (CRP). For CPRD the median for years since first CV disease event was used as dates since first diagnosis might be subject to changes in coding and be potentially unreliable. For the Veterans Affairs, HUNT3 and Karelia, use of antithrombotic drugs was unavailable, however as these drugs are available as over the counter drugs and recommended for all patients with atherosclerotic CV disease it was assumed that all patients used this.

This approach to missing data in a prognostic study would lead to a more conservative estimate of the discrimination (as not all model parameters are available for risk stratification, discrimination decreases). However, the effect on calibration would be more limited assuming roughly similar prevalence of systematic missing risk factors in derivation and validation data.

Methods S5. Lifetable estimation

The lifetable approach combines the baseline hazards with the cause specific cox models to obtain competing risk adjusted estimates for the risk of recurrent CV events and non-CV death for individual patients. Using the individual risk factor levels the linear predictors (LP_recurrent cv_ and LP_non-CV ­death_) for both endpoints can be estimated using the Supplementary Table 5. These can be combined with the age-specific 1-year interval survival probabilities for a patients remaining life years up to the age of 90 for both endpoints.

**Step 1.** The 1-year interval specific survival probabilities for the specific patient can be estimated using: S_recurrent CV events_ (t) $=S_{0}{(t)}^{exp(LP recurrent cv events)}$for recurrent CV events and S_non-CV death_(t) = $S_{0}\left( t \right)^{\exp\left( LP treated non CV death \right)}$ for non-CV death. Here *t* represents the ages for the remaining life years for the patient up till age 90.

**Step 2.** Combining the 1-year interval survival probability for recurrent CV events and non-CV death yields the overall probability of surviving the interval event free.

**Step 3.** The cumulative event free survival can be estimated by multiplying the 1-year interval probabilities successively.

**Step 4.** The probability of any event occurring in a specific 1-year interval can be estimated. These can be combined with the proportion of recurrent CV events and proportional of non-CV death in that same interval. Combining these respective proportions with the probability of any event occurring yields the interval risk for both respective endpoints, thus ensuring that competing risks are accounted for, that the total proportion of risk aligns and maintaining the relationship between overall survival and event-specific risks.

These steps fill in the lifetable for the patient with a specific risk profile. For example, a male patient aged 65 with arbitrary values of 0 for LP_recurrent cv_ and LP_non-CV ­death_ would have the following result:

| Age | Interval event-free survival (%) | Cumulative survival (%) | Annual CV event risk (%) | Annual non-CV death (%) | Cumulative CV events (%) | Cumulative non-CV death (%) |
| --- | --- | --- | --- | --- | --- | --- |
| 65 | 97.4 | 97.4 | 1.3 | 1.3 | 1.3 | 1.3 |
| 66 | 97.2 | 94.7 | 1.4 | 1.4 | 2.7 | 2.7 |
| 67 | 97.1 | 91.9 | 1.4 | 1.4 | 4.1 | 4.1 |
| 68 | 96.9 | 89.0 | 1.4 | 1.4 | 5.5 | 5.5 |
| 69 | 96.7 | 86.1 | 1.5 | 1.5 | 6.9 | 6.9 |
| 70 | 96.5 | 83.1 | 1.5 | 1.5 | 8.4 | 8.4 |
| 71 | 96.3 | 80.1 | 1.5 | 1.5 | 10.0 | 10.0 |
| 72 | 96.1 | 76.9 | 1.6 | 1.6 | 11.5 | 11.5 |
| 73 | 95.8 | 73.7 | 1.6 | 1.6 | 13.1 | 13.1 |
| 74 | 95.6 | 70.5 | 1.6 | 1.6 | 14.8 | 14.8 |
| The table contains example values of the lifetable calculations for a male patient with a linear predictor sum of 0 for both respective endpoints. Age indicates the current age for a patient and all interval probabilities refer to the probability of surviving that specific age interval event free. CV, cardiovascular. | | | | | | |

Column 1 (*Interval event free survival*) is the results of Steps 1 and 2 (see above). It contains the probability of surviving a specific 1-year interval (e.g., turning 66) free of both recurrent CV events and non-CV death. Column 2 (*cumulative survival*) is the results of Step 3. It contains the cumulative survival of all the interval risks up to each remaining life year. For example, the probability of surviving op till 67 years without any event, given that one has just turned 65 is (2-year survival) is 94.7% (row 2). The CV-free life expectancy as defined as the age where the cumulative survival probability drops below 50% can be read from the lifetable. Columns 3 and 4 (*annual CV event risk; annual non-CV death risk)* contain the interval specific risk for either endpoint. For example, column 3 row 1 shows a 1.3% risk of experiencing a recurrent CV event in the life year from 65 to 66. Columns 5 and 6 (*cumulative CV events; cumulative non-CV death)* hold the cumulative risk of columns 3 and 4. These should be read as a 1-year risk for row 1, a 2-year risk for row 2 etc. When doing this for 40-year-old it can yield up to a 50-year risk.

The precise calculations underlying this can be seen in the SMART-REACH2 function on the [GitHub](https://github.com/JorisHolt/SMARTREACH2) repository.

Methods S6. Calibration plots

Calibration plots were made accounting for competing risks. First, predicted risks were ordered from low to high. Risks were rounded to no decimals (e.g., 38.1% was rounded to 38%) and all unique predicted risks in the data were extracted. At each of the unique risk estimates the number of observed events was estimated using the *cuminc* function R, obtaining both the observed incidence at that particular risk estimate as well as the standard error for the estimation. Following sequencing along all predicted risks a loess regression was performed using the number of observations at each unique risk as weights. Similarly, the confidence intervals were obtained by using the standard errors to obtain 95% confidence intervals, to which the same loess regression was applied to obtain smoothed estimates. As some unique risks have few observations a dynamic window was used that included the observations from the unique risk estimate and those within ±1% from that unique risk. This window was expanded dynamically (by multiplying the window of 1% with 1.5 etc.) until it contained at least two primary events and two other outcomes (either competing events or censored cases). For visualization purposes, the predicted risks were truncated at the 99th percentile to avoid unstable estimates in regions with very few individuals.

The background histogram in the plots shows the distribution of predicted risks in the population, allowing readers to assess the density of predictions across different risk levels.

This approach was verified in the UCC-SMART dataset by estimating the number of patients at each unique risk that were within the bounds of the 95% confidence interval corresponding to that risk. A span value of 0.5 was used for the complete data and a span of 0.75 was used for subgroups to account for smaller sample sizes.

Methods S7. Recalibration

The SMART-REACH2 model can be recalibrated to other data sources using sex-specific expected-observed (EO) ratio’s for both recurrent CV events and non-CV death. One of two approaches can be taken, firstly, if the new data is part of one of the risk regions to which the model has been recalibrated already these regional recalibration factors can be used. If for some reason these factors are deemed not representative for the new data (e.g., specialized care settings), the model can be recalibrated to the new data at hand using the following procedure.

*Data requirements*
The data should consist of patients that align with the intended use of the model. This specifically means patients with established stable atherosclerotic CV disease (Supplemental table 1 and 2) aged between 40-90 years in a stable phase (e.g., 6 weeks after their last event). Patients with an expected short life expectancy (e.g., terminal illness) or for whom long-term prognostication is not relevant for other reasons should be removed prior.

A hard requirement is the completeness and reliability of the follow-up and outcome data for both endpoints. This should be used to make the competing event indicator (CV event: if a recurrent CV event occurred; Non-CV death: if the patient died without a prior CV event; Censored: if neither occurred). The follow-up by definition in that case is the follow-up from inclusion to recurrent CV events or end of study. Preferable all predictors should be present. Missing data should be addressed using imputation incorporating the outcome data as Nelson-Aalen estimator. In case of systematic missingness (a variable is completely missing) either median imputation (using the median level in the derivation data) or assumptions have to be made (e.g., when use of antithrombotic drug use is unavailable the assumption that all patients are using it is can be made). Using this data the following steps should be taken:

1. A time-horizon for risk predictions needs to be selected. Considerations are 1) that longer follow-up for recalibration aligns more with the intended use of the model (i.e., when long term risk predictions are required, these should be well calibrated) and 2) the estimates should be reliable at that risk horizon. This second requirement can be addressed by taken the floored rounded value of the 75^th^ percentile of follow-up (e.g., when the 75^th^ percentile is 2.4 years one should consider using a 2-year risk for recalibration).
2. Estimating risk for recurrent CV events and non-CV death at the selected time horizon using the SMART-REACH2 model.
3. Estimate the mean predicted risk for both endpoints for men and women separately for those patients who have a predicted risk at the selected time horizon. As the model predict risk up till 90 years old, only patients with age 80 or younger have a 10-year predicted risk; thus the mean risk should be estimated for those patients where a risk has been estimated.
4. Estimate the mean predicted risk for both endpoints for men and women separately for those patients who have a predicted risk at the selected time horizon (e.g. using the ‘cuminc’ function in R). As the model predict risk up till 90 years old only patients with age 80 or younger have a 10-year predicted risk; thus the mean risk should be estimated for those patients where a risk has been estimated. Neglecting to take this into account in Steps 3 and 4 will lead to an overestimation of the observed risk as elderly patients contribute to the cumulative incidence but not to the predicted risk.
5. Estimate the sex-specific expected observed ratio per outcome by dividing the predicted by Awomen for the respective end points by taking *lineair predictor – log(eo)*
6. Calibration for both endpoints should be reassessed to assess whether recalibration was successful. If this is not the case steps 3-5 should be repeated, using the risk estimates following the first round of recalibration. The new EO-ratios should be multiplied with the prior EO-ratios and the product of these should be used for recalibration by taking *linear predictor – log(eo_prior_ ­­_*­ ­­_ eo_new_).* Step 6 should be repeated to assess recalibration success.

Methods S8. Estimation of treatment benefits

The SMART-REACH2 model can be combined with causal treatment effects from trials and meta-analyses to estimate potential treatment benefits.

*General approach*
The risk of recurrent CV events and non-CV death should be estimated for the current risk profile and treatments. Causal (relative) treatment effects (such as hazard ratio; HR) for the treatment of interest (i.e., the one under consideration) have to be obtained for both endpoints predicted by the model (i.e., recurrent CV events should be defined in the same or similar way for the causal effect as well as the SMART-REACH2 model). These estimates can be combined with the lifetable approach to obtain treatment benefits (Methods S3. Lifetable estimation). Step 1 from those outlined in that section needs to be replaced with the step below.

1. The treatment effect needs to be applied to the linear predictor for the respective endpoint to which it applies. As the linear predictor is on the log-scale the logarithm of the HR needs to be used: *Linear predictor (LP) with treatment =* *LP without treatment + log(HR).* Where *LP treated* is the new linear predictor including treatment effect and HR is the hazard ratio for the treatment.

The absolute risk reductions due to treatment initiation and/or intensification can be estimated for the different prediction horizons (e.g., 10-year risk) by subtracting the treated 10-year risk from the untreated 10-year risk. The lifetime benefit can be estimated by subtracting the untreated CV-free life expectancy from the treated CV-free life expectancy (i.e., the age at which the survival probability is 0.5). These steps only apply to treatments that are being considered for that patient (i.e., not current treatments). Treatments that a patient is receiving already should be part of the linear predictor for the current risk profile. This is especially relevant for treatments whose effects is not fully mediated by the predictors in the model (e.g., SGLT2-inhibitors). For treatments whose effects are mediated by the predictors in the models (e.g., non-HDL cholesterol and statins) the current risk factor levels can be used). When estimating the effect of more than one therapy at once the HR’s for the respective treatments have to be multiplied and their effects are assumed to be conditionally independent.

*Example calculations*
In the current paper the effect of lowering systolic blood pressure and LDL-cholesterol is illustrated. For blood pressure lowering a hazard ratio of 0.80 for recurrent CV events per 10 mmHg reduction and no effect on non-CV death was used. For LDL cholesterol a HR of 0.78 for recurrent CV events per 1 mmol/L lowering was used. For the illustrated patient cases a reduction from 150 mmHg to 135 mmHg and 1.8 mmol/L to 1.4 mmol/L was used ^2,37,38^.

*Treatment estimations past the maximum prediction horizon*
Median life expectancy is estimated as the age at which the disease-free survival probability is equal to 50%. For patients with favourable risk profiles, this point of median survival can theoretically lie beyond the maximum age up to which the model estimates risk (i.e., 90 years). As such, the outlined approach approach for estimating median CV-free life expectancy cannot be used. For these patients the gain in CV-free life expectancy due to treatment is based on the survival probability at age 90. For example, for an off-treatment survival probability of 55% at age 90 (end of the prediction horizon) the gain in life expectancy is based on the survival relative to the treated survival. When for this patient the treated survival probability reaches 55% (e.g., at age 87), the difference in age between the untreated and treated survival at the predicted maximum untreated survival is taken to be the gain in CV-free life expectancy, in this case 3 years.

Methods S9. Assumptions of lifetable for risk estimation and treatment effects

| Assumption | Explanation |
| --- | --- |
| Lifetime prediction | |
| Proportionality of predictors | The cause-specific hazard models assume that the effects of the predictors are proportional over age; that is, the estimated coefficients reflect the constant effect of each risk predictor across the complete age range of the population. For the current models this means that the coefficients should remain stable over the ages in the cohort (40-90 years; e.g, the hazard ratio for diabetes mellitus should be similar at age 50 and age 80). Alternatively phrased, that the average effect for a certain predictor is a good approximation of its effect over the entire age range. This assumption is tested by visual assessment of the scaled Schoenfeld residuals over time. In case of violations interactions with the time scale are considered. In case of no or very minimal violations the model is kept as parsimonious as possible. |
| Linearity of the predictor-outcome relation | The cause-specific hazard models assume a log-linear relationship between the linear predictor and the hazard rate. However, the relationship between individual continuous predictors and the outcome can be non-linear. Such non-linearity can be accommodated within the linear predictor framework by applying transformations to predictors or adding polynomial terms. The appropriate transformations were assessed using Akaike Information Criterion (AIC). When a specific transformation or addition of polynomial terms decreased the AIC by ≥ 2, the predictor was incorporated into the model in this modified form. When multiple transformations or polynomials met this criterion, the option with the lowest AIC was selected for the final model. |
| Stationarity of baseline hazards | The cause specific hazard models require a baseline hazard usually estimated by the model itself. This baseline hazard in this case is held by the lifetable. This lifetable contains the probability of surviving each 1-year age interval (e.g., probability of turning 61 when having turned 60). This baseline hazard is estimated separately for each endpoint for a patient with sex-specific mean values for the continuous predictors and all binary predictors set to 0. This baseline hazard is the same for all patients and the linear predictor is applied to it. The assumption is that the baseline hazard also represents the hazard for patients that in the future will enter a specific interval. For example, it assumes that for a patient who is 60 now the probability of turning 61 is the same as for a patient who is 50 now and will only be 60 in 10-years (i.e., the probability of turning 61 is the same for either given that they have survived up till 60 years). |
| Treatment effects | |
| Equal relative treatment effect | When estimating treatment benefits the assumption is that there is no heterogeneity in treatment effect. As such the marginal treatment effect as reported in trials and/or meta-analyses is taken to be the effect for all patients. This assumption is made since subgroup analyses from trials and meta-analyses mostly have not identified significant differences in relative treatment effects between eligible patients with varying characteristics ^37,38^. |
| Constant treatment effect over time | It is assumed that these relative treatment effects remain constant over time, so that therapy benefits continue to accrue over lifetime exposure. In other words, it is assumed that the hazard ratios found in actual trials or meta-analyses are equal to hazard ratios that would have been found in trials with lifelong follow-up. |
| Additive benefits | It is assumed that benefits of individual therapies are independently of each other when used in conjunction. For treatment effects on as used on the log scale (i.e., log hazard ratio), this means additive independence, and for the treatment effects on non-log scale (i.e., hazard ratios) this means multiplicative independence). This is only applicable in the absence of reliable estimates of efficacy for combined interventions. In case there is evidence for dependence both treatment effects could be amended |
| AIC, Akaike’s information criterion. | |

**Supplementary Tables**

Supplementary Table 1. Definitions of atherosclerotic CV disease at baseline in UCC-SMART

| CV disease subtype | Definition |
| --- | --- |
| Coronary artery disease | Defined as history of as angina pectoris with documented stenosis, myocardial infarction, or coronary revascularization (coronary bypass surgery or coronary angioplasty) |
| Cerebrovascular disease | Defined as history of a transient ischaemic attack, cerebral infarction, amaurosis fugax or retinal infarction, or a history of carotid surgery; |
| Peripheral artery disease | Defined as history of symptomatic and documented obstruction of distal arteries of the leg or a history of vascular surgery of the leg (percutaneous transluminal angioplasty, bypass, or amputation); |
| Aortic abdominal aneurysm | Defined as history of presence of a supra- or infrarenal aneurysm of the aorta (distal aortic anteroposterior diameter ≥3 cm, measured at baseline examination with ultrasonography) or a history of AAA surgery. |
| Abbreviations: cm, centimetre; AAA, aortic abdominal aneurysm | |

Supplementary Table 2. Definition of history of vascular disease or diabetes mellitus at baseline

| History of established CV disease | ICD-10 codes |
| --- | --- |
| History of coronary artery disease | I20.0;I21-I23; I25. |
| History of cerebrovascular disease | I61;I63;I64;I63;I65;I66;I67.2; |
| History of peripheral artery disease | I70.2, I70.20, I.70.21, I70.22, I70.23, I70.24, I70.25, I70.26, I70.29 |
| History of abdominal aortic aneurysm | I71.3, I71.4 |
| Established risk factors |  |
| Diabetes Mellitus (type 1/2) | E10-E11 |
| Abbreviations: ICD, international classification of disease. Ideally the definition of CV disease at baseline were those above. These were adapted/restricted in certain countries based on local practices. | |

Supplementary Table 3. Endpoint definitions

| Fatal cardiovascular disease | |
| --- | --- |
| Endpoints included | **ICD10-codes** |
| Hypertensive disease | I10-16 |
| Ischemic heart disease | I20-25 |
| Arrhythmias, heart failure | I46-52 |
| Cerebrovascular disease | I60-69 |
| Atherosclerosis/AAA | I70-73 |
| Sudden death and death within 24h of symptom onset | R96.0-96.1 |
| *Excluding the following* |  |
| Myocarditis, unspecified | I51.4 |
| Subarachnoid haemorrhage | I60 |
| Subdural haemorrhage | I62 |
| Cerebral aneurysm | I67.1 |
| Cerebral arteritis | I68.2 |
| Moyamoya | I67.5 |
| Non-fatal events |  |
| Endpoints included | **ICD10-codes** |
| Non-fatal myocardial infarction | I21-I23 |
| Non-fatal stroke | I60-69 |
| *Excluding the following* |  |
| Subarachnoid haemorrhage | I60 |
| Subdural haemorrhage | I62 |
| Cerebral aneurysm | I67.1 |
| Cerebral arteritis | I68.2 |
| Moyamoya | I67.5 |
| Abbreviations: ICD, international classification of disease; h, hours; AAA, aortic abdominal aneurysm. Endpoint definitions depend on cohort availability and country specific practices regarding use of certain ICD-10 codes (e.g., use of ICD-10 code I69 for hospitalisations for other reasons in patients with a baseline history of stroke in Norway). | |

Supplementary Table 4. Proportion of missing data in predictor variables and outcomes in UCC-SMART cohort

| Predictor | Missing (%) |
| --- | --- |
| Sex | 0% |
| Age | 0% |
| Current smoking | 0.51% |
| Systolic blood pressure | 1.10% |
| Diabetes mellitus | 0.02% |
| History of coronary artery disease | 0% |
| History of cerebrovascular disease | 0% |
| History of peripheral artery disease | 0% |
| History of abdominal aortic aneurysm | 0% |
| C-reactive protein | 3.23% |
| Years since first atherosclerotic CV disease diagnosis | 1.53% |
| Total cholesterol | 0.41% |
| HDL-cholesterol | 0.57% |
| Creatinine | 0.38% |
| Using aspirin or equivalent | 0% |
| Non-CV death | 0% |
| Recurrent CV events | 0% |
| Follow-up time for non-CV death | 0% |
| Follow-up time for recurrent CV events | 0% |
| Abbreviations: CV, cardiovascular; HDL, high density lipoprotein | |

Supplementary Table 5. Penalized sex-specific hazard ratios for recurrent CV events and non-CV death per standard deviation increase

|  | Model for recurrent CV events | | Model for non-CV death | |
| --- | --- | --- | --- | --- |
|  | Men | Women | Men | Women |
| Age |  |  |  |  |
| Age as timescale | * | | * | |
| Age at entry (years) | 0.72 [0.65-0.81] | 0.78 [0.63-0.97] | 0.51 [0.45-0.58] | 0.42 [0.33-0.53] |
| Current smoking | 1.40 [1.25-1.57] | 1.67 [1.34-2.07] | 1.49 [1.30-1.72] | 1.62 [1.28-2.05] |
| Systolic blood pressure (mmHg) | 1.04 [0.99-1.09] | 1.00 [0.90-1.11] | 1.01 [0.95-1.07] | 1.01 [0.90-1.13] |
| Non-HDL cholesterol (mmol/L)^a^ | 1.10 [1.04-1.16] | 1.05 [0.95-1.17] | 0.93 [0.87-0.99] | 0.82 [0.73-0.92] |
| Diabetes mellitus | 1.39 [1.23-1.56] | 1.52 [1.21-1.91] | 1.19 [1.03-1.38] | 1.32 [1.01-1.72] |
| History of coronary artery disease | 1.27 [1.10-1.47] | 1.57 [1.18-2.09] | 0.84 [0.70-1.01] | 0.94 [0.66-1.35] |
| History of cerebrovascular disease | 1.36 [1.19-1.56] | 1.81 [1.38-2.37] | 1.11 [0.94-1.31] | 1.16 [0.82-1.64] |
| History of peripheral artery disease | 1.25 [1.09-1.44] | 1.52 [1.16-2.00] | 1.36 [1.15-1.61] | 1.41 [1.01-1.98] |
| History of abdominal aortic aneurysm | 1.41 [1.22-1.64] | 1.59 [1.09-2.33] | 1.17 [0.97-1.41] | 1.57 [1.01-2.44] |
| Years since first atherosclerotic CV diagnosis^b,c^ | 1.12 [1.06-1.18]^b^ | 1.06 [0.96-1.17]^b^ | 1.17 [1.10-1.25]^c^ | 1.13 [1.02-1.26]^c^ |
| eGFR (mL/min/1.73m^2^)^b^ | 0.88 [0.83-0.94] | 0.85 [0.76-0.96] | 1.06 [0.99-1.15] | 1.01 [0.88-1.14] |
| C-reactive protein (mg/L)^a^ | 1.21 [1.14-1.28] | 1.23 [1.10-1.38] | 1.21 [1.12-1.29] | 1.22 [1.08-1.38] |
| Estimates represent the cause-specific associations between predictors and the respective outcomes. Hazard ratios are displayed for 1 standard deviation increase in the predictor values using sex-specific standard deviations. Confidence intervals are 95% confidence intervals estimated using a pooled covariance structure. Hazard-ratios and confidence intervals cannot be interpreted causally as these are biased using ridge penalization and therefor do not represent the usual values optimized using maximum likelihood estimation, as such the confidence interval does not represent true uncertainty in the coefficient estimation. * Age is incorporate as the underlying timescale of the model, and its effect is captured by the baseline hazard as such coefficients can be counterintuitive and should not be interpreted. Transformations for continuous variables are a. log transformation, b, squared transformation, and c, square root transformation. The square root transformation for Years since first atherosclerotic CV disease diagnosis is for non-CV death. Use anticoagulants was included as an offset with hazard ratio of 0.81 for the models with recurrent CV events. Abbreviations: CV, cardiovascular; HDL, high density lipoprotein; eGFR, estimated glomerular filtration rate. | | | | |

Supplementary Table 6. Model coefficients for respective endpoints per sex

|  | Men | | Women | |
| --- | --- | --- | --- | --- |
|  | **Recurrent CV** | **Non-CV death** | **Recurrent CV** | **Non-CV death** |
| Age |  |  |  |  |
| Age as timescale | * | | * | |
| Age at entry (years) | -0.324061 | -0.670561 | -0.247403 | -0.872887 |
| Current smoking | 0.337445 | 0.401107 | 0.510218 | 0.480229 |
| Systolic blood pressure | 0.039264 | 0.005069 | -0.002440 | 0.006242 |
| Diabetes mellitus | 0.327418 | 0.175527 | 0.419332 | 0.277386 |
| History of coronary artery disease | 0.241155 | -0.172170 | 0.450037 | -0.057874 |
| History of cerebrovascular disease | 0.307973 | 0.103424 | 0.594076 | 0.147620 |
| History of peripheral artery disease | 0.226948 | 0.307799 | 0.419664 | 0.343675 |
| History of abdominal aortic aneurysm | 0.346496 | 0.157130 | 0.463149 | 0.451213 |
| Years since first atherosclerotic CV disease diagnosis (years) | 0.337353 | - | 0.651165 | - |
| Years since first atherosclerotic CV disease diagnosis (years, squared) | -0.224587 | - | -0.591588 | - |
| Years since first atherosclerotic CV disease diagnosis (years, square root) | - | 0.159750 | - | 0.124516 |
| Non-HDL cholesterol (log, mmol/L) | 0.092652 | - | 0.052014 | - |
| Non-HDL cholesterol (mmol/L) | - | -0.075039 | - | -0.200458 |
| eGFR, (mL/min/1.73 m^2^) | -1.126501 | -1.017214 | -0.678423 | -0.473617 |
| eGFR (squared, mL/min/1.73 m^2^) | 1.002846 | 1.079151 | 0.519648 | 0.479040 |
| C-reactive protein (log, mg/L) | 0.189486 | 0.187284 | 0.207823 | 0.199951 |
| Use of antithrobotics | -0.210721 | - | -0.210721 | - |
| * The effect of age as a timescale is incorporate in the baseline hazard, available in the supplementary material. Coefficients are on the log scale per 1 standard deviation increase for the continuous variables. The standard deviations are sex specific. For the binary predictor variables, the coefficients represent the presence of the predictor. Coefficients cannot be interpreted causally as these are the result of ridge penalization and therefor biased compared to unpenalized coefficients. Lambda values for penalization as a result from the 5-fold cross validation were λcv range 0.677-0.995 (median 0.846) and λnon-cv death range 0.538-0.718 (median 0.635) for men and λcv 1.772-2.009 (median 1.850) and λnon-cv death range 2.689-3.720 (median 3.059) for women. Abbreviations: CV, cardiovascular; HDL, high density lipoprotein; eGFR, estimated glomerular filtration rate. | | | | |

Supplementary Table 7. Sex-specific means and standard deviations used for scaling and centring

|  | Men | | Women | |
| --- | --- | --- | --- | --- |
| Predictor | **Mean** | **SD** | **Mean** | **SD** |
| Age (years) | 61.51 | 9.16 | 60.95 | 9.96 |
| Systolic blood pressure (mmHg) | 138.83 | 18.94 | 138.60 | 20.29 |
| Years since first atherosclerotic CV disease diagnosis (years) | 4.07 | 6.91 | 2.95 | 6.22 |
| Years since first atherosclerotic CV disease diagnosis (years, squared) | 64.30 | 157.98 | 47.35 | 161.31 |
| Years since first atherosclerotic CV disease diagnosis (years, square root) | 1.25 | 1.58 | 0.97 | 1.42 |
| Non-HDL cholesterol (log, mmol/L) | 1.21 | 0.32 | 1.24 | 0.34 |
| Non-HDL cholesterol (mmol/L) | 3.51 | 1.13 | 3.66 | 1.22 |
| eGFR (mL/min/1.73 m^2^) | 77.36 | 17.06 | 74.30 | 17.57 |
| eGFR (squared, mL/min/1.73 m^2^) | 6275.75 | 2522.34 | 5828.68 | 2554.60 |
| C-reactive protein (log, mg/L) | 0.61 | 1.14 | 0.77 | 1.14 |
| Values represent the mean and standard deviation for both sexes in the derivation data, which were used to scale and centre the predictor values. Abbreviations: SD, standard deviation; CV, cardiovascular; HDL, high-density lipoprotein; eGFR, estimated glomerular filtration rate. | | | | |

Supplementary Table 8. Interval specific baseline survival for respective endpoints per sex

|  | Men | | Women | |
| --- | --- | --- | --- | --- |
| Age | **Survival for recurrent CV events** | **Survival for non-CV death** | **Survival for recurrent CV events** | **Survival for non-CV death** |
| 40 | 0.99540522 | 0.99983732 | 0.99960895 | 0.99996064 |
| 41 | 0.99530913 | 0.99981005 | 0.99956489 | 0.99995282 |
| 42 | 0.99520344 | 0.99977820 | 0.99951588 | 0.99994345 |
| 43 | 0.99508718 | 0.99974102 | 0.99946134 | 0.99993221 |
| 44 | 0.99495930 | 0.99969760 | 0.99940066 | 0.99991873 |
| 45 | 0.99481864 | 0.99964691 | 0.99933315 | 0.99990258 |
| 46 | 0.99466391 | 0.99958771 | 0.99925803 | 0.99988322 |
| 47 | 0.99449373 | 0.99951859 | 0.99917444 | 0.99986002 |
| 48 | 0.99430653 | 0.99943789 | 0.99908144 | 0.99983220 |
| 49 | 0.99410062 | 0.99934365 | 0.99897797 | 0.99979885 |
| 50 | 0.99387412 | 0.99923362 | 0.99886284 | 0.99975887 |
| 51 | 0.99362499 | 0.99910514 | 0.99873474 | 0.99971095 |
| 52 | 0.99335096 | 0.99895511 | 0.99859221 | 0.99965350 |
| 53 | 0.99304953 | 0.99877994 | 0.99843362 | 0.99958464 |
| 54 | 0.99271798 | 0.99857540 | 0.99825716 | 0.99950210 |
| 55 | 0.99235328 | 0.99833658 | 0.99806083 | 0.99940315 |
| 56 | 0.99195214 | 0.99805771 | 0.99784239 | 0.99928453 |
| 57 | 0.99151089 | 0.99773209 | 0.99759933 | 0.99914234 |
| 58 | 0.99102554 | 0.99735188 | 0.99732890 | 0.99897189 |
| 59 | 0.99049167 | 0.99690793 | 0.99702800 | 0.99876757 |
| 60 | 0.98990445 | 0.99638956 | 0.99669320 | 0.99852264 |
| 61 | 0.98925852 | 0.99578428 | 0.99632069 | 0.99822904 |
| 62 | 0.98854803 | 0.99507753 | 0.99590622 | 0.99787709 |
| 63 | 0.98776653 | 0.99425229 | 0.99544506 | 0.99745519 |
| 64 | 0.98690691 | 0.99328870 | 0.99493194 | 0.99694944 |
| 65 | 0.98596136 | 0.99216358 | 0.99436103 | 0.99634319 |
| 66 | 0.98492130 | 0.99084982 | 0.99372580 | 0.99561645 |
| 67 | 0.98377728 | 0.98931583 | 0.99301901 | 0.99474528 |
| 68 | 0.98251891 | 0.98752466 | 0.99223260 | 0.99370099 |
| 69 | 0.98113476 | 0.98543321 | 0.99135760 | 0.99244915 |
| 70 | 0.97961225 | 0.98299113 | 0.99038404 | 0.99094852 |
| 71 | 0.97793757 | 0.98013965 | 0.98930080 | 0.98914967 |
| 72 | 0.97609548 | 0.97681012 | 0.98809554 | 0.98699333 |
| 73 | 0.97406927 | 0.97292241 | 0.98675451 | 0.98440844 |
| 74 | 0.97184052 | 0.96838294 | 0.98526240 | 0.98130984 |
| 75 | 0.96938900 | 0.96308245 | 0.98360221 | 0.97759544 |
| 76 | 0.96669244 | 0.95689334 | 0.98175501 | 0.97314286 |
| 77 | 0.96372634 | 0.94966664 | 0.97969971 | 0.96780539 |
| 78 | 0.96046376 | 0.94122841 | 0.97741289 | 0.96140717 |
| 79 | 0.95687506 | 0.93137555 | 0.97486845 | 0.95373740 |
| 80 | 0.95292766 | 0.91987088 | 0.97203739 | 0.94454338 |
| 81 | 0.94858569 | 0.90643748 | 0.96888740 | 0.93352218 |
| 82 | 0.94380971 | 0.89075203 | 0.96538257 | 0.92031067 |
| 83 | 0.93855634 | 0.87243695 | 0.96148292 | 0.90447356 |
| 84 | 0.93277787 | 0.85105142 | 0.95714398 | 0.88548906 |
| 85 | 0.92642181 | 0.82608066 | 0.95231626 | 0.86273166 |
| 86 | 0.91943042 | 0.79692364 | 0.94694469 | 0.83545155 |
| 87 | 0.91174020 | 0.76287854 | 0.94096802 | 0.80274992 |
| 88 | 0.90328130 | 0.72312588 | 0.93431807 | 0.76354931 |
| 89 | 0.89397688 | 0.67670882 | 0.92691901 | 0.71655814 |
| Values represent the probability of surviving the 1-year interval without experiencing the respective endpoint, not accounting for the other endpoint. For the first row, for example, the value in column for “Survival for recurrent CV events” represents the probability of turning 41 years old for a 40-year-old. Abbreviations: CV, cardiovascular. | | | | |

Supplementary Table 9. Expected observed ratios for recalibration to the respective risk regions.

|  | Men | | Women | |
| --- | --- | --- | --- | --- |
|  | **Recurrent CV events** | **Non-CV death** | **Recurrent CV events** | **Non-CV death** |
| European low-risk region | 0.937731 | 0.751485 | 1.025123 | 0.556574 |
| European moderate-risk region | 0.805820 | 1.472000 | 0.644238 | 0.909291 |
| European high-risk region | 0.419713 | 1.129708 | 0.456383 | 1.186722 |
| European very-high-risk region | 0.288743 | 1.004505 | 0.240195 | 0.716020 |
| North America | 0.581047 | 0.811941 | 0.500844 | 0.383206 |
| Latin America | 0.534811 | 0.987646 | 0.388429 | 0.385655 |
| Asia + Middle East | 0.398453 | 0.887095 | 0.320499 | 0.457972 |
| Australia | 0.844303 | 0.805417 | 0.917705 | 0.550384 |
| Japan | 0.851833 | 1.390348 | 0.793415 | 1.194257 |
| For the low-risk region HUNT3 was used for recalibration at 10-years of follow-up. For the moderate-risk region SWEDEHEART was used for recalibration at 10 years of follow-up. For the high-risk region. the combination of REACH and EUROASPIRE V was used at 2 year of follow-up. For the very-high-risk region the combination of REACH EUROASPIRE V and SURF-CHDII Serbia was used at 2 year of follow-up. For North America, Asia + Middle East, Latin America. Australia. and Japan the REACH data at 2-years of follow-up was used. The VA was recalibrated using VA data at 3-years of follow-up. Abbreviations: CV, cardiovascular. | | | | |

**Supplementary Figures**

## Supplementary Figure 1. Origin of European data sources in relation to the four European risk regions

Map of Europe with ESC risk region definitions and cohort origins. Assignment of countries to each risk region were based on the SCORE2 definitions

## Supplementary Figure 2. Steps for risk estimation using SMART-REACH2

GitHub available [here](https://github.com/JorisHolt/SMARTREACH2). Abbreviations: CAD, coronary artery disease; non-HDL-C, high-density lipoprotein cholesterol; eGFR, estimated glomerular filtration rate; CRP, C-reactive protein, cardiovascular disease; LP, linear predictor.

## Supplementary Figure 3. Baseline hazards for recurrent CV events and non-CV death in women

Both plots show the conditional 1-year interval survival probability (i.e., probability of turning 41 given that one has turned 40) as estimated by the cause-specific Cox model (in red). The exponential model fitted to these probabilities is in blue. This line is weighted for the number of observations contributing to each 1-year interval over the complete follow-up (histogram for scale). The baseline hazard used for the cox model are those estimated by the exponential model (blue dots). Equations for smoothed lines for both endpoints: S_CV_=1−( 5.46886 ×10^−6)⋅^e^−0.106744⋅age^; S_non cv_ _death_=1−(2.79307 ×10^−8^)⋅e^−0.181267⋅age^

## Supplementary Figure 4. Baseline hazards for recurrent CV events and non-CV death in men

Both plots show the conditional 1-year interval survival probability (i.e., probability of turning 41 given that one has turned 40) as estimated by the cause-specific Cox model (in red). The exponential model fitted to these probabilities is in blue. This line is weighted for the number of observations contributing to each 1-year interval over the complete follow-up (histogram for scale). The baseline hazard used for the cox model are those estimated by the exponential model (blue dots). Equations for smoothed lines for both endpoints: S_CV_=1−(2.12744 ×10^−5^)⋅e^-0.095270 ⋅age^; S_non cv_ _death_=1−(3.3027×10^−7^)⋅e^−0.15499⋅age^

## Supplementary Figure 5. Internal validation in UCC-SMART

Calibration plots display predicted risk (x-axis) against observed risk (y-axis), with 95% confidence intervals. Histograms show the distribution of predicted risks. For internal validation the EO-ratio’s for UCC-SMART were used as estimated at 10-years of follow-up for display purposes only as to account for the effects of ridge regression. These were 0.8641711 for recurrent CV events and 0.8410980 for non-CV death in men and 0.8448131 for recurrent CV events and 0.6635622 for non-CV death in women. Patient counts may be lower than the total cohort population as individuals turning >90 during the prediction horizon are excluded (e.g., 89-year-olds cannot contribute to 2-year risk estimates).

## Supplementary Figure 6. Calibration for non-CV death in external validation cohorts from the European low and moderate-risk regions

Calibration plots display predicted risk (x-axis) against observed risk (y-axis), with 95% confidence intervals. Histograms show the distribution of predicted risks. Cohort sizes here may be lower than the total cohort population listed in Table 2 as risk can only be estimated up to age 90 (e.g., 89-year-olds can have 1-year risk but no risks for 2-year or higher and are consequently dropped at the horizon for validation). Abbreviations: CV, cardiovascular; EA-V, EUROASPIRE-V. Cohorts belonging to the low-risk region are highlighted in green and cohorts belonging to the moderate region are highlighted in yellow. Abbreviations: EA-V, EUROASPIRE V.

## Supplementary Figure 7. Calibration for non-CV death in external validation cohorts from European high- and very-high-risk regions

Calibration plots display predicted risk (x-axis) against observed risk (y-axis), with 95% confidence intervals. Histograms show the distribution of predicted risks. Cohort sizes here may be lower than the total cohort population listed in Table 2 as risk can only be estimated up to age 90 (e.g., 89-year-olds can have 1-year risk but no risks for 2-year or higher and are consequently dropped at the horizon for validation). Cohorts belonging to the high-risk region are highlighted in orange and cohorts belonging to the very-high-risk region are highlighted in red. The combination of high-risk cohorts consists of REACH, EUROASPIRE-V and Bialystok POLASPIRE, the combination for the very-high-risk region consists of REACH, EUROASPIRE and SURF-CHD.

## Supplementary Figure 8. Calibration for non-CV death in external validation cohorts outside of Europe

Calibration plots display predicted risk (x-axis) against observed risk (y-axis), with 95% confidence intervals. Histograms show the distribution of predicted risks. Cohort sizes here may be lower than the total cohort population listed in Table 2 as risk can only be estimated up to age 90 (e.g., 89-year-olds can have 1-year risk but no risks for 2-year or higher and are consequently dropped at the horizon for validation). Classification according to European risk regions do not apply and as such are not highlighted in the title.

## Supplementary Figure 9. Age-specific calibration for recurrent CV events in external validation cohorts from the European low- and moderate-risk regions

The figure displays the average predicted (blue) and observed risk (yellow) at each specific age at the time of enrolment for those patients in whom the risk could be estimated. For example, HUNT3 is displayed at 10-years of risk, consequently, only patients aged 40-80 are displayed, as no 10-year risk can be estimated for patients who are older. The light grey section represents the total number of individuals in that particular 1-year age bin, the darker grey the number of events in that bin during the follow-up. The histograms are scaled to the total population of each cohort and should not be compared. Abbreviations: CV, cardiovascular; EA-V, EUROASPIRE-V. Cohorts belonging to the low-risk region are highlighted in green and cohorts belonging to the moderate region are highlighted in yellow.

## Supplementary Figure 10. Age-specific calibration for recurrent CV events in external validation cohorts from the European high- and very-high-risk regions

The figure displays the average predicted (blue) and observed risk (yellow) at each specific age at the time of enrolment for those patients in whom the risk could be estimated. For example, Estonian Biobank is displayed at 10-years of risk, consequently, only patients aged 40-80 are displayed, as no 10-year risk can be estimated for patients who are older. The light grey section represents the total number of individuals in that particular 1-year age bin, the darker grey the number of events in that bin during the follow-up. The histograms are scaled to the total population of each cohort and should not be compared. Cohorts belonging to the high-risk region are highlighted in orange and cohorts belonging to the very-high-risk region are highlighted in red. The combination of high-risk cohorts consists of REACH, EUROASPIRE-V and Bialystok POLASPIRE, the combination for the very-high-risk region consists of REACH, EUROASPIRE and SURF-CHD.

## Supplementary Figure 11. Age-specific calibration for recurrent CV events in external validation cohorts outside of Europe

The figure displays the average predicted (blue) and observed risk (yellow) at each specific age at the time of enrolment for those patients in whom the risk could be estimated. For example, Veterans Affairs is displayed at 3-years of risk, consequently, only patients aged 40-87 are displayed, as no 3-year risk can be estimated for patients who are older. The light grey section represents the total number of individuals in that particular 1-year age bin, the darker grey the number of events in that bin during the follow-up. The histograms are scaled to the total population of each cohort and should not be compared. Classification according to European risk regions do not apply and as such are not highlighted in the title. The bracket in the Veterans Affairs represents the age at which people become eligible for healthcare outside of the Veterans Affairs, at which point events may be missed. The grey line represents the extrapolation of the event rates over time based on those aged 62 or younger, which may be viewed as the expected number of events had outcome ascertainment been complete.

.

## Supplementary Figure 12. External validation in the Veterans Affairs cohort for patients eligible only for health care through the Veterans Affairs

The top row represents the calibration plot on the full population, including those patients eligible to receive care from Medicare/Medicaid. The observed proportion drops in line with the age of eligibility for alternative healthcare access. The bracket in the Veterans Affairs represents the age at which people become eligible for healthcare outside of the Veterans Affairs, at which point events may be missed. The grey line represents the extrapolation of the event rates over time based on those aged 62 or younger, which may be viewed as the expected number of events had outcome ascertainment been complete. In the bottom row the calibration is presented for those individuals eligible for care through the VA alone. The issue of miscalibration is resolved. The difference in the height of the histogram are due to difference in the reference population used for scaling. In the top row this is full population, in the bottom row this it that proportion of the population aged 62 year or younger, giving the impression that the age bracket of 45 is larger than in the top row, which is not the case. The C-statistic for the restricted Veterans Affairs would be 0.65 [0.65- 0.65] overall (n = 103,439), 0.64 [0.64-0.65] for men (n=95,861), and 0.72 [0.70-0.73] for women (n = 7,578)

## Supplementary Figure 13. Discrimination for recurrent CV events following regional recalibration for the European low-risk region

Forest plots display the cohort specific and region-specific pooled discrimination as measured by C-statistics for recurrent CV events adjusted for the risk of non-CV death. Cohort sizes here may be lower than the total cohort population listed in Table 2 as risk can only be estimated up to age 90 (e.g., 89-year-olds can have 1-year risk but no risks for 2-year or higher and are consequently dropped at the horizon for validation). Abbreviations: EA-V, EUROASPIRE V; CI confidence interval; I^2^, heterogeneity.

## Supplementary Figure 14. Discrimination for recurrent CV events following regional recalibration for the European moderate-risk region

Forest plots display the cohort specific and region-specific pooled discrimination as measured by C-statistics for recurrent CV events adjusted for the risk of non-CV death. Cohort sizes here may be lower than the total cohort population listed in Table 2 as risk can only be estimated up to age 90 (e.g., 89-year-olds can have 1-year risk but no risks for 2-year or higher and are consequently dropped at the horizon for validation). Abbreviations: EA-V, EUROASPIRE V; CI confidence interval; I^2^, heterogeneity.

## Supplementary Figure 15. Discrimination for recurrent CV events following regional recalibration for the European high- and very-high-risk region

Forest plots display the cohort specific and region-specific pooled discrimination as measured by C-statistics for recurrent CV events adjusted for the risk of non-CV death. Cohort sizes here may be lower than the total cohort population listed in Table 2 as risk can only be estimated up to age 90 (e.g., 89-year-olds can have 1-year risk but no risks for 2-year or higher and are consequently dropped at the horizon for validation). Abbreviations: EA-V, EUROASPIRE V; CI confidence interval; I^2^, heterogeneity.

## Supplementary Figure 16. Discrimination for recurrent CV events following recalibration for the non-European risk regions

Forest plots display the cohort specific and region-specific pooled discrimination as measured by C-statistics for recurrent CV events adjusted for the risk of non-CV death. Cohort sizes here may be lower than the total cohort population listed in Table 2 as risk can only be estimated up to age 90 (e.g., 89-year-olds can have 1-year risk but no risks for 2-year or higher and are consequently dropped at the horizon for validation). Abbreviations: CI confidence interval; I^2^, heterogeneity.

## Supplementary Figure 17. Discrimination for non-CV death following regional recalibration

Forest plots display the cohort and pooled region-specific and overall discrimination as measured by C-statistics for non-CV death adjusted for the risk of recurrent CV. Cohort sizes here may be lower than the total cohort population listed in Table 2 as risk can only be estimated up to age 90 (e.g., 89-year-olds can have 1-year risk but no risks for 2-year or higher and are consequently dropped at the horizon for validation). Abbreviations: EA-V, EUROASPIRE V; CI confidence interval; EU, Europe; I^2^, heterogeneity.

## Supplementary Figure 18. Discrimination for recurrent CV events following regional recalibration according to sex

Forest plots display the pooled discrimination as measured by C-statistics for recurrent CV events adjusted for the risk of non-CV death according to sex and European vs. non-European origin. In each cohort C-statistics are estimated at the same follow-up duration as for the complete cohort. Cohort sizes here may be lower than the total cohort population listed in Table 2 as risk can only be estimated up to age 90 (e.g., 89-year-olds can have 1-year risk but no risks for 2-year or higher and are consequently dropped at the horizon for validation). Abbreviations: CI confidence interval; EU, Europe.

## Supplementary Figure 19. Discrimination for non-CV death following regional recalibration according to sex

Forest plots display the pooled discrimination as measured by C-statistics for non-CV death adjusted for the risk of recurrent CV events according to sex and European vs. non-European origin. In each cohort C-statistics are estimated at the same follow-up duration as for the complete cohort. Cohort sizes here may be lower than the total cohort population listed in Table 2 as risk can only be estimated up to age 90 (e.g., 89-year-olds can have 1-year risk but no risks for 2-year or higher and are consequently dropped at the horizon for validation). Abbreviations: CI confidence interval; EU, Europe.

## Supplementary Figure 20. Calibration for recurrent CV events in women following regional recalibration

Calibration plots display predicted risk (x-axis) against observed risk (y-axis), with 95% confidence intervals. Histograms show the distribution of predicted risks. Cohort sizes here may be lower than the total cohort population listed in Table 2 as risk can only be estimated up to age 90 (e.g., 89-year-olds can have 1-year risk but no risks for 2-year or higher and are consequently dropped at the horizon for validation). Cohorts belonging to the low-risk region are highlighted in green and cohorts belonging to the moderate region are highlighted in yellow, cohorts belonging to the high-risk region are highlighted in orange and cohorts belonging to the very-high-risk region are highlighted in red. Classification according to European risk regions do not apply and as such are not highlighted in the title. The combination of high-risk cohorts consists of REACH, EUROASPIRE-V and POLASPIRE, the combination for the very-high-risk region consists of REACH, EUROASPIRE and SURF-CHD. Abbreviations: CV, cardiovascular.

## Supplementary Figure 21. Calibration for recurrent CV events in men following regional recalibration

Calibration plots display predicted risk (x-axis) against observed risk (y-axis), with 95% confidence intervals. Histograms show the distribution of predicted risks. Cohort sizes here may be lower than the total cohort population listed in Table 2 as risk can only be estimated up to age 90 (e.g., 89-year-olds can have 1-year risk but no risks for 2-year or higher and are consequently dropped at the horizon for validation). The combination of high-risk cohorts consists of REACH, EUROASPIRE-V and POLASPIRE, the combination for the very-high-risk region consists of REACH, EUROASPIRE and SURF-CHD. Abbreviations: CV, cardiovascular.

## Supplementary Figure 22. Calibration for non-CV death in men following regional recalibration

Calibration plots display predicted risk (x-axis) against observed risk (y-axis), with 95% confidence intervals. Histograms show the distribution of predicted risks. Cohort sizes here may be lower than the total cohort population listed in Table 2 as risk can only be estimated up to age 90 (e.g., 89-year-olds can have 1-year risk but no risks for 2-year or higher and are consequently dropped at the horizon for validation). Cohorts belonging to the low-risk region are highlighted in green and cohorts belonging to the moderate region are highlighted in yellow, cohorts belonging to the high-risk region are highlighted in orange and cohorts belonging to the very-high-risk region are highlighted in red. Classification according to European risk regions do not apply and as such are not highlighted in the title. The combination of high-risk cohorts consists of REACH, EUROASPIRE-V and POLASPIRE, the combination for the very-high-risk region consists of REACH, EUROASPIRE and SURF-CHD. Abbreviations: CV, cardiovascular.

## Supplementary Figure 23. Calibration for non-CV death in women following regional recalibration

Calibration plots display predicted risk (x-axis) against observed risk (y-axis), with 95% confidence intervals. Histograms show the distribution of predicted risks. Cohort sizes here may be lower than the total cohort population listed in Table 2 as risk can only be estimated up to age 90 (e.g., 89-year-olds can have 1-year risk but no risks for 2-year or higher and are consequently dropped at the horizon for validation). Cohorts belonging to the low-risk region are highlighted in green and cohorts belonging to the moderate region are highlighted in yellow, cohorts belonging to the high-risk region are highlighted in orange and cohorts belonging to the very-high-risk region are highlighted in red. Classification according to European risk regions do not apply and as such are not highlighted in the title. The combination of high-risk cohorts consists of REACH, EUROASPIRE-V and POLASPIRE, the combination for the very-high-risk region consists of REACH, EUROASPIRE and SURF-CHD. Abbreviations: CV, cardiovascular.

## Supplementary Figure 24. Comparison of the SMART-REACH and SMART-REACH2 model

The figure displays the calibration, C-statistics and decision curves for the SMART-REACH and SMART-REACH2 model in HUNT3 (low risk; green) and SWEDEHEART (moderate risk; yellow). The C-statistics in SWEDEHEART are those with all predictors used. The asterix (*) represents the C-statistic when removing patients with heart failure and atrial fibrillation which are used by the SMART-REACH model as predictors, given an advantage to SMART-REACH in a coronary artery disease only cohort. In HUNT3, with general population prevalence for these predictors SMART-REACH2 maintained higher C-statistic. The decision curves are for both models using all predictors, displaying higher clinical utility for SMART-REACH2 in spite of the disadvantage in predictor specificity in SWEDEHEART.

## Supplementary Figure 25. Discrimination for recurrent CV events according CV disease subtypes following regional recalibration

Forest plots display the pooled discrimination as measured by C-statistics for non-CV death adjusted for the risk of recurrent CV events for different subtypes of atherosclerotic CV disease and European vs. non-European origin. These atherosclerotic CV disease subtypes are not exclusive (e.g., coronary artery disease contains all patients with coronary artery disease, not just patients with only coronary artery disease). In each cohort C-statistics are estimated at the same follow-up duration as for the overall. Cohort sizes here may be lower than the total cohort population listed in Table 2 as risk can only be estimated up to age 90 (e.g., 89-year-olds can have 1-year risk but no risks for 2-year or higher and are consequently dropped at the horizon for validation). Abbreviations: CV, cardiovascular; CI confidence interval; EU, Europe

## Supplementary Figure 26. Discrimination for non-CV death according CV disease subtypes following regional recalibration

Forest plots display the pooled discrimination as measured by C-statistics for non-CV death adjusted for the risk of recurrent CV events for different subtypes of atherosclerotic CV disease and European vs. non-European origin. These atherosclerotic CV disease subtypes are not exclusive (e.g., coronary artery disease contains all patients with coronary artery disease, not just patients with only coronary artery disease). In each cohort C-statistics are estimated at the same follow-up duration as for the overall. Cohort sizes here may be lower than the total cohort population listed in Table 2 as risk can only be estimated up to age 90 (e.g., 89-year-olds can have 1-year risk but no risks for 2-year or higher and are consequently dropped at the horizon for validation). Abbreviations: CV, cardiovascular; CI confidence interval; EU, Europe

## Supplementary Figure 27. Calibration of recurrent CV events according to CV disease subtypes in HUNT3

Calibration plots display predicted risk (x-axis) against observed risk (y-axis), with 95% confidence intervals. Histograms show the distribution of predicted risks. For plots “with” a CV subtype all patients with that specific subtype are included. For plots “only” with a CV subtype only patients with a single CV subtype are included. Cohort sizes here may be lower than the total cohort population listed in Table 2 as risk can only be estimated up to age 90 (e.g., 89-year-olds can have 1-year risk but no risks for 2-year or higher and are consequently dropped at the horizon for validation). Abbreviations: CAD, coronary artery disease; CeVD, cerebrovascular disease; PAD, peripheral artery disease.

## Supplementary Figure 28. Calibration for recurrent CV events and non-CV death in the NHSE SDE cohort during the COVID-19 pandemic

 Calibration plots display predicted risk (x-axis) against observed risk (y-axis), with 95% confidence intervals. Histograms show the distribution of predicted risks. Cohort sizes here may be lower than the total cohort population listed in Table 2 as risk can only be estimated up to age 90 (e.g., 89-year-olds can have 1-year risk but no risks for 2-year or higher and are consequently dropped at the horizon for validation). Cohorts belonging to the low-risk region are highlighted in green and cohorts belonging to the moderate region are highlighted in yellow. Abbreviations: CV, cardiovascular.
